# Hypoxia Decreases Nitrogen Removal in Coastal Marine Sediments

**DOI:** 10.1021/acs.est.5c04877

**Published:** 2026-03-30

**Authors:** Jing Sun, Xingyu Yang, Liuqian Yu, Qiong Zhang, Charmaine C. M. Yung, Jiying Li

**Affiliations:** † Department of Ocean Science, 58207 The Hong Kong University of Science and Technology, Clear Water Bay, Kowloon, Hong Kong SAR 999077, P. R. China; ‡ Center for Ocean Research in Hong Kong and Macau, Kowloon, Hong Kong SAR 999077, P. R. China; § Earth, Ocean and Atmospheric Sciences Thrust, The Hong Kong University of Science and Technology (Guangzhou), Guangzhou, Guangdong 511400, P.R. China

**Keywords:** coastal oceans, sediments, nitrogen cycling, hypoxia, Pearl River Estuary region

## Abstract

Hypoxia impacts sediment nitrogen (N) cycling, yet its effects remain poorly constrained and exhibit strong cross-system inconsistency. We investigate these mechanisms through observations and mass-balance analysis of the Pearl River Estuary region, a typical estuarine coastal system under diverse environmental regimes. Our results show that low-oxygen conditions in the bottom waters increased sediment ammonium efflux. Under well-oxygenated bottom waters, 75% of the ammonium released from organic matter was oxidized within the sediments, with the remaining 25% entering the water column. Under low-oxygen conditions, ammonium efflux increased to 55% of that from organic matter, and only 45% of remineralized ammonium was oxidized through nitrification. With reduced sediment nitrification under hypoxia, denitrification decreased, removing only 53% of remineralized organic N in sediments compared to 76% under high bottom-oxygen conditions. This is because denitrification depends heavily on nitrate supply by nitrification, while nitrate concentrations in bottom waters were too low to support adequate nitrate influx to the sediments. Using a mass-balance analysis, we further demonstrate that this mechanism operates in many coastal systems with low-to-moderate nitrate levels. Thus, bottom-water hypoxia likely enhances sediment N recycling and reduces N removal on a broader scale, creating amplifying feedback to eutrophication in coastal N-limiting systems.

## Graphical abstract

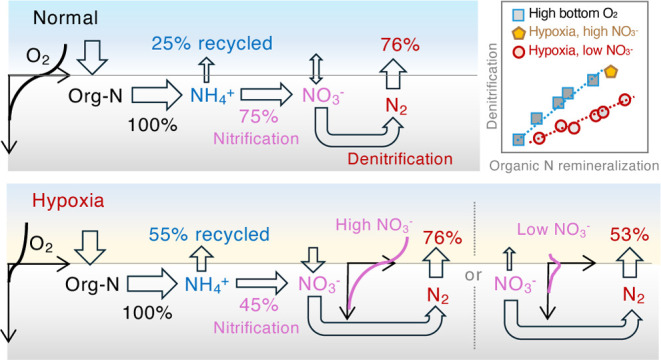

## Introduction

Nitrogen (N) frequently limits primary production in marine ecosystems. In coastal oceans, the availability of N in the water column is regulated by external inputs, N fixation, anaerobic removal, and N recycling from sediments.^1–4^ In sediments, the remineralization of organic matter produces ammonium, which can be oxidized to nitrate under oxic conditions; both species can be recycled back into the water column.^5,6^ Conversely, anoxic sediments can produce N_2_ via denitrification and anaerobic oxidation of ammonium (anammox), removing fixed N from the system.^1,7^ The balance between sediment N recycling and removal is regulated by oxygen availability.^1,8^ As observed in many coastal systems,^9–12^ bottom-water hypoxia (dissolved oxygen less than ∼100 μmol L^–1^) can promote nitrate reduction, thereby enhancing N removal in organic-rich sediments. However, in some other systems, low oxygen can hinder nitrification (oxidation of ammonium to nitrate), thereby decreasing coupled nitrification–denitrification and N removal.^13–16^ Mechanisms leading to this variability across systems remain poorly quantified but are crucial for predicting whether sediments’ feedback to bottom-water hypoxia amplifies or mitigates eutrophication. Resolving the mechanisms of the cross-system variability is critical because coastal hypoxia, which affects biogeochemical cycles and threatens aquatic life, is becoming increasingly frequent globally.^17–19^

Constraining the impact of hypoxia on sediment N recycling and removal is technically challenging, particularly for teasing apart the specific effects of low oxygen from those of covarying substrates. For example, comparing N fluxes and rates of N transformations in sediments from different sites or across seasons with varying oxygen levels can be problematic. This is because low-oxygen conditions in the field are often driven by strong stratification of the water column and high rates of organic matter remineralization (i.e., in areas of high primary productivity and sedimentation).^20–22^ Consequently, the observed variations in the rates of N transformations, such as denitrification, could be driven primarily by greater organic matter availability rather than by low oxygen level itself.^8,23^ While experiments using sediments from the same location for incubations under different oxygen conditions may elucidate hypoxia impacts, measured potential rates may misrepresent reality, as modifying substrate availabilities (e.g., NH_4_^+^ and NO_3_^–^) can obscure their natural responses to changing oxygen levels. In-situ techniques and intact core incubations that allow for extrapolation of in situ rates may provide the most accurate representations of in situ processes,^13,24^ but such data are scarce due to the complexity of the experiments and the need for larger numbers of sediment cores. This makes large-scale studies difficult, especially when investigating spatial variability under different environmental regimes.

In this paper, we use the Pearl River Estuary and its offshore shelf as a model system to investigate how bottom-water hypoxia affects sediment N recycling and removal. The Pearl River Estuary region exhibits strong spatial variability in the physiochemistry of the water column and sediments. Beyond salinity, dissolved nutrient concentrations vary strongly, driven by the gradient between high-nutrient terrestrial and low-nutrient marine sources.^25^ Inorganic N in the water column varies by several orders of magnitude, decreasing from upstream to the offshore shelf waters.^22,25^ The nutrient variability and hydrodynamic conditions have led to higher primary productivity within the estuary compared to the shelf and the development of summer hypoxia across a large area at the estuary mouth.^20,22,26^ Sediments in the region are largely anoxic, characteristic of organic-rich coastal environments.^20^ These sediments are hotspots of nitrogen removal, sustained by higher organic matter deposition, and contribute substantially to the system’s N budget.^27^ The spatial variabilities in the bottom-water nitrate and oxygen levels, as well as organic matter sedimentation and remineralization rates, provide a unique setting to investigate how N recycling and removal under various regimes. Using a mass-balance approach, we examine the effects of substrate availability and oxygen concentrations on the sediment N cycling in the region, particularly how bottom-water oxygen levels moderate the responses of N fluxes and rates to organic matter remineralization. The analysis further reconciles inconsistencies observed across systems and reveals the mechanisms that determine whether sediment feedback amplifies or mitigates eutrophication.^17,28^

## Methods

### The Study Area and Sampling Methods

The Pearl River Estuary receives freshwater from the Pearl River Basin (4.4 × 10^5^ km^2^).^29^ Heavy seasonal rainfall (annual precipitation of 1200–2200 mm concentrated in coastal areas from April and September), combined with intensive agriculture, urban and industrial activities in the densely populated Pearl River Delta, leads to high nutrient loading in the river system.^29^ The estuary is mesotrophic to eutrophic, with chlorophyll a concentration ranging 1.1–12 μg L^–1^ and total phosphorus concentration decreasing from ∼2.0–2.2 μmol L^–1^ in the estuary (A03 to A08) to ∼1.1–1.2 μmol L^–1^ offshore (2A01b to 2A02).^30^ Bottom hypoxia develops in the summer (typically June to August) within nearshore waters (10–20 m depth),^20,31^ driven by strong water-column stratification that inhibits oxygen exchange from the surface.^20,21^ While hypoxia in the region is seasonal, its intensity has increased over decades.^31,32^

Samples were collected from the Pearl River Estuary and its adjacent shelf (9–63 m depth; Figure 1 and Table S1) during the summer of 2021 across an area of a wide range of salinity, bottom-water oxygen and nitrate concentrations, and sediment organic matter content and oxygen uptake rates (see Results and Discussion later). The physicochemical parameters of the water column were measured using a Sea-Bird SBE19plus Conductivity–Temperature–Depth (CTD) probe and were reported in our previous studies.^20,30,33^ Sediment cores were collected with overlying waters of 15–20 cm using a gravity corer (86 mm ID and 60 cm length). Overlying waters from approximately 0.5–1 cm above the sediment surface were collected, filtered through 0.2-μm cellulose acetate membrane filters, and preserved for analysis of dissolved substances. Sediment oxygen profiles were measured onboard using Clark-type microelectrodes (Unisense). Separate intact sediment cores were sectioned onboard in a glovebag filled with N_2_, and porewaters were extracted using Rhizon Samplers of 0.1-μm pore size. The samples were processed onboard within a few hours of collection. Porewater, overlying water, and solid sediment samples were stored frozen (−20 °C) before analysis.

**Figure 1:**
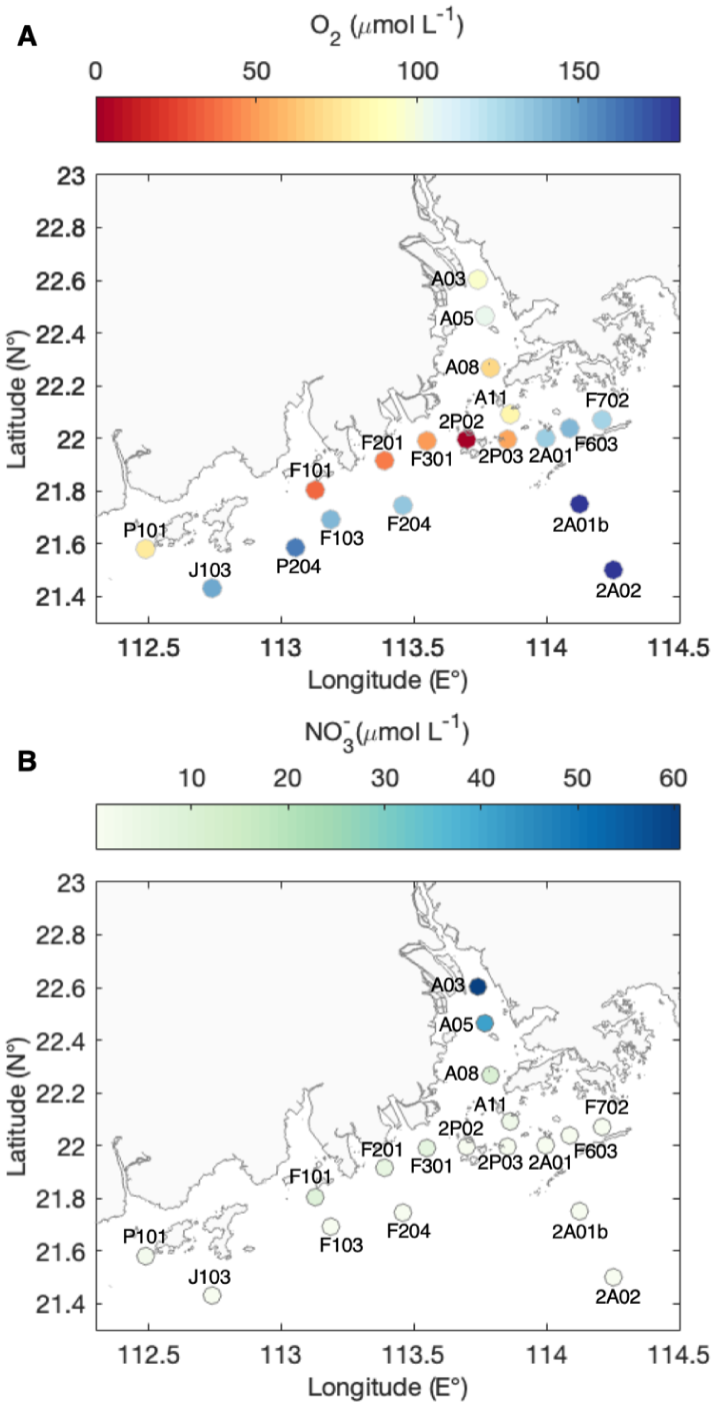
Sampling locations and (A) bottom-water oxygen concentrations and (B) bottom-water nitrate concentrations in the Pearl River Estuary region. Detailed spatial maps of additional environmental parameters for these stations can be found in Sun et al. 2024 and 2025.^20,31^

### Analysis of Sediment Physical and Chemical Properties

Concentrations of nitrate and nitrite in porewater and overlying waters were measured using ion chromatography connected to UV spectrometry at a wavelength of 220 nm. Ammonium concentrations were measured using the standard phenate method (APHA-4500-NH3) on a microplate reader.^34^ Sediment particulate organic carbon (POC) and particulate nitrogen (PN) were measured using an elemental analyzer (Euro EA3000, Eurovector) following acid washing (0.5 N of hydrochloric acid overnight) to remove particulate inorganic carbon.^35^ Note that this treatment also removes some soluble or volatilized organic matter, leading to underestimation of POC and PN.^36^ Sediment water content was determined by weight loss after drying. Porosity was calculated from water content and dried density measured by a pycnometer. The sediments were dated by measuring the radioactivity of ^210^Pb using a gamma spectrometer, and sediment accumulation rates (g cm^–2^ yr^–1^) were calculated using a constant rate of supply (CRS) model.^37^ All geochemical measurements, except for ^210^Pb dating, were performed in triplicate. Reported values are reported as means ± standard deviation.

### Estimation of Fluxes across the Sediment–Water Interface

Sediment oxygen uptake (SOU), which is the downward flux of oxygen into the sediments, was determined using whole core incubations. Methods are detailed in (Supporting Information SI.1) and results are reported in our previous study.^20^ Note that SOU was measured to estimate the rate of sediment organic carbon remineralization (see later), because oxygen is consumed either directly in the oxidation of organic carbon or in the oxidation of the reduced species (e.g., Fe^2+^, Mn^2+^, H_2_S) produced by anaerobic carbon remineralization, leading to a 1C:1O_2_ stoichiometry. However, SOU can be limited by oxygen availability when bottom-water oxygen concentrations fall below ∼47–100 μmol L^–1^,^39^ and reduced species from anaerobic carbon remineralization might not be entirely oxidized, leading to underestimation of organic carbon remineralization rates using SOU. To address this issue, we bubbled sufficient air into the overlying waters before the incubation to ensure that oxygen uptake was not limited by low oxygen concentration during the incubation. This approach overestimates the in situ SOU at those hypoxic sites, but it provides a more accurate estimate of organic carbon mineralization, better serving the purpose of this study (see later). The prebubbling treatment was applied only to SOU measurement; all other measurements were performed on separate, intact cores and were thus unaffected.

We estimate the molecular diffusive fluxes (*F* ^diff^) of oxygen, ammonium, and nitrate across the sediment-water interface using Fick’s law of diffusion:^39^

1 $$F_{i}^{\mathrm{diff}}=-\varphi D_{i}^{s}\frac{dC_{i}}{dz}$$

where *i* represents the species of interest (O_2_, NH_4_^+^ or NO_3_^–^); φ is the porosity of the surface sediments (0–0.5 cm); $D_{i}^{s}$ is the molecular diffusion coefficient corrected for sediment tortuosity;^39^ *C*_*i*_ is the concentration in the porewater; *z* is the sediment depth (zero at the interface and positive downward; positive flux means downward flux). Molecular diffusive flux only accounts for a portion of the total flux, which is also contributed by other processes, especially bioirrigation and bottom-water turbulence in coastal oceans.^40^ For oxygen, we can estimate the bioirrigated flux $\left(F_{O_{2}}^{\mathrm{irr}}\right)$ as the difference between the measured SOU and the sediment oxygen diffusive flux $\left(F_{O_{2}}^{\mathrm{diff}}\right)$ :

2 $$F_{O_{2}}^{\mathrm{irr}}=\mathrm{SOU}-F_{O_{2}}^{\mathrm{diff}}$$

Flux due to bioirrigation can be considered as enhanced diffusivity.^41,42^ Hence, we can estimate the bioirrigated flux of other species $\left(F_{i}^{\mathrm{irr}}\right)$ considering bioirrigation coefficient proportional to the molecular diffusion coefficient:^43^

3 $$F_{i}^{\mathrm{irr}}=\left(\frac{F_{O_{2}}^{\mathrm{irr}}}{F_{O_{2}}^{\mathrm{diff}}}\right)\times F_{i}^{\mathrm{diff}}$$

Details of the calculations and uncertainties are described in SI.2. The effects of the uncertainties on the results will be discussed later. Total flux across the sediment-water interface can be calculated as

4 $$F_{i}=F_{i}^{\mathrm{diff}}+F_{i}^{\mathrm{irr}}$$

### Rate Estimates Using a Mass-Balance Approach

We use a mass-balance approach to estimate the rates of various N cycling processes, particularly nitrification and N removal via the production of N_2_. Ammonium in sediments is produced from organic matter remineralization (Table S1). A proportion of the NH_4_^+^ released from organic matter is oxidized via nitrification, while dissimilatory nitrate reduction (DNRA) converts nitrate back to ammonium. For simplicity, we use “nitrification” to refer to the net ammonium oxidation resulting from nitrification and DNRA. Some NH_4_^+^ is buried in the sediments through adsorption on the solid particles.^44^ These processes together result in net production of NH_4_^+^ within the sediment and flux of NH_4_^+^ into the overlying water:

5 $$F_{{\mathrm{NH}}_{4}^{+}}=F_{{\mathrm{NH}}_{4}^{+}}^{\mathrm{diff}}+F_{{\mathrm{NH}}_{4}^{+}}^{\mathrm{irr}}=-\left(\left(1-\alpha_{\mathrm{nitrif.}}\right)\frac{F_{C}}{r_{C:N}}-F_{{\mathrm{NH}}_{4}^{+}}^{\mathrm{bur}}\right))$$

Here, α_nitrif._ is nitrification efficiency, defined as the proportion of NH_4_^+^ being nitrified in the total amount of NH_4_^+^ remineralized from organic matter in the sediments. *F*_C_ is the rate of organic carbon remineralization (all rates are positive numbers hereafter) following a remineralization C:N ratio (*r*_C:N_). $F_{{\mathrm{NH}}_{4}^{+}}^{\mathrm{bur}}$ is the burial flux of NH_4_^+^ in the sediments.

For simplicity, the remineralization C:N ratio (*r*_C:N_) was approximated using the C:N ratio in the surface sediments (Table S2). However, sediment C:N ratios may differ from those of remineralized organic matter due to mixed sources of organic matter with varying C:N ratios and reactivities. For instance, vascular-plant materials have higher C:N ratios and are less readily mineralized than autochthonous planktonic organic matter.^45^ The preferential degradation of fresher organic matter is consistent with the general increase of C:N ratio with depth at some sites (see results later). For sediments exhibiting a generally consistent, nonfluctuating trend of increasing C:N ratio with depth, the remineralization C:N ratios (*r*_C:N_) were corrected as the ratios of the POC and PN changing rates $\left(\frac{d\mathrm{POC}}{dz}:\frac{d\mathrm{PN}}{dz}\right)$ . We also treat the C:N variability as a source of uncertainty in our subsequent estimates using *r*_C:N_, since the variability might simply reflect historical changes in organic matter composition.

Ammonium burial flux $F_{{\mathrm{NH}}_{4}^{+}}^{\mathrm{bur}}$ was estimated at the bottom of the core as (details in SI.3):

6 $$F_{{\mathrm{NH}}_{4}^{+}}^{\mathrm{bur}}=C_{{\mathrm{NH}}_{4}^{+}}K_{{\mathrm{NH}}_{4}^{+}}\frac{\varphi }{\left(1-\varphi \right)}\frac{1}{\rho }F_{\mathrm{sed}}^{\mathrm{bur}}$$

where $K_{{\mathrm{NH}}_{4}^{+}}$ is the unitless adsorption coefficient of NH_4_^+^ in sediments.^44^ ρ is the density of dry sediments, and $F_{\mathrm{sed}}^{\mathrm{bur}}$ is the sediment accumulation rate (e.g., in g cm^–2^ y^–1^).

Remineralization of organic carbon and oxidation of NH_4_^+^ consume oxygen (Table S1):

7 $$\mathrm{SOU}={\mathrm{SOU}}_{C}+{\mathrm{SOU}}_{\mathrm{nitrif.}}=\left(1-\gamma_{\mathrm{denitrif.}}\right)F_{C}+\frac{2}{r_{C:N}}\alpha_{\mathrm{nitrif.}}F_{C}$$

where SOU_C_ and SOU_nitrif._ are SOU due to carbon remineralization (1C:1O_2_) and nitrification (1NH_4_^+^:2O_2_), respectively (Table S1); γ_denitrif._ is the proportion of organic matter remineralization contributed by denitrification. Here, denitrification is excluded when relating organic matter remineralization to SOU because the product of denitrification, N_2_, does not consume oxygen. Other anaerobic carbon degradation pathways are accounted for in the 1C:1O_2_ stoichiometry, as oxygen is the ultimate acceptor of electrons from organic carbon (see SI.1).^40^ A 1NH_4_^+^:2O_2_ stoichiometry is assumed for nitrification, but incomplete nitrification (production of NO_2_^–^) may occur (see SI.4 for calculations and discussion later for uncertainties).^46^ We also assume that the primary source of sediment NH_4_^+^ is the degradation of organic matter and external sources are negligible (see Results later showing sediments are net source of NH_4_^+^ rather than receiving NH_4_^+^ from water column).

While *r*_C:N_, $F_{{\mathrm{NH}}_{4}^{+}}$ , $F_{{\mathrm{NH}}_{4}^{+}}^{\mathrm{bur}}$ and SOU are either directly measured or calculated, the two eqs (eqs 5 and 7) together still have three variables unknown, *F*_C_, α_nitrif._ and γ_denitrif_. To solve the equations, we further consider the mass balance concerning the inorganic N in the sediments:

8 $$\frac{1}{r_{C:N}}F_{C}+F_{{\mathrm{NO}}_{3}^{-}}+F_{{\mathrm{NH}}_{4}^{+}}-F_{{\mathrm{NH}}_{4}^{+}}^{\mathrm{bur}}-F_{N_{2}}=0$$

The first term of the left-hand side of the equation is the production of NH_4_^+^ from the remineralization of organic matter; $F_{N_{2}}$ is the removal of N via the production of N_2_ (or N_2_O) by denitrification and anammox. Anammox is of minor importance in the region (<7% of total N_2_ production in the estuary and <5% offshore),^27,47^ confirmed by isotope tracing experiments that also exclude other anaerobic ammonium oxidation processes, such as that coupled to sulfate reduction (sulfammox).^48^ Thus, we assume N_2_ to be produced primarily by denitrification $\left(F_{N_{2}}=F_{\mathrm{denitrif.}}\right)$ , the rate of which can be linked to organic carbon remineralization using the C:N stoichiometry of the reaction (Table S1):

9 $$F_{N_{2}}=F_{\mathrm{denitrif.}}=\frac{4}{5}\gamma_{\mathrm{denitrif.}}F_{C}$$

Therefore, eq 8 becomes

10 $$\frac{1}{r_{C:N}}F_{C}+F_{{\mathrm{NO}}_{3}^{-}}+F_{{\mathrm{NH}}_{4}^{+}}-F_{{\mathrm{NH}}_{4}^{+}}^{\mathrm{bur}}-\frac{4}{5}\gamma_{\mathrm{denitrif.}}F_{C}=0$$

We can then solve the series of linear equations, eqs 5, 7, and 10, for the unknown variables *F*_C_, α_nitrif._ and γ_denitrif._ (see SI.4 for solutions). Rates of N_2_ production (denitrification) can then be estimated using eq 9. Rates of nitrification can be estimated as

11 $$F_{\mathrm{nitrif.}}=\frac{1}{r_{C:N}}\alpha_{\mathrm{nitrif.}}F_{C}$$

Flux and rate values are reported as mean ± standard deviation.

## Results and Discussion

### Spatial Variability of Oxygen and Nitrate in the Water Column

Like many other estuarine systems, the Pearl River Estuary region exhibits strong spatial variability in the physiochemistry of the water column (Table 1, Figure 1). Driven by high primary productivity and strong summer stratification, low-oxygen conditions are observed in the bottom waters across a wide area, including the mainstem and the mouth of the estuary, as well as the shallow shelf waters (Figure 1A),^20^ consistent with observations from recent years.^21,31,32^ In addition to the salinity gradient typical of a freshwater–seawater transition (Table 1), nitrate concentrations in the bottom waters decrease from 61 μmol L^–1^ upstream (i.e., at A03) to near the detection limit (∼0.3 μmol L^–1^) offshore (Figure 1B), likely resulting from the higher nutrient input near terrestrial sources.^25^

**Table 1:** Sampling Site, Total Water Depth (Depth), Surface Water Salinity, Bottom O_2_ Concentration, Bottom NO_3_^–^ Concentration, Sediment Oxygen Uptake (SOU), Sediment Organic Carbon Remineralization Rate (*F*_C_), Diffusive Fluxes of NH_4_^+^ and NO_3_^–^ across the Sediment–Water Interface ( $F_{{\mathrm{NH}}_{4}^{+}}^{\mathrm{diff}}$ and $F_{{\mathrm{NO}}_{3}^{-}}^{\mathrm{diff}}$ ), Burial Flux of NH_4_^+^ $\left(F_{{\mathrm{NH}}_{4}^{+}}^{\mathrm{bur}}\right)$ , Sediment Nitrification Rate (*F*_nitrif._), Sediment N_2_ Production Rate ( $F_{N_{2}}$ ; mostly from denitrification), the Proportion of NH_4_^+^ Produced from Organic Matter Remineralization that is Nitrified (α__s‑nitrif_._), the Proportion of Organic Matter Remineralization Contributed by Denitrification (γ_denitrif_), and the ratio of nitrification and denitrification rates (*F*_nitrif._ ^a^

Sites	Depth (m)	Salinity (ppt)	Bottom O_2_ (μmol L^–1^)	Bottom NO_3_ (μmol L^–1^)	SOU (mmol m^–2^ d^–1^)	*F*_C_ (mmol m^–2^ d^–1^)	$F_{{\mathrm{NH}}_{4}^{+}}^{\mathrm{diff}}$ (mmol m^–2^ d^–1^)	$F_{{\mathrm{NO}}_{3}^{-}}^{\mathrm{diff}}$ (mmol m^–2^ d^–1^)	$F_{{\mathrm{NH}}_{4}^{+}}^{\mathrm{bur}}$ (μmol m^–2^ d^–1^)	*F*_nitrif._ (mmol m^–2^ d^–1^)	$F_{N_{2}}$ (mmol m^–2^ d^–1^)	α__nitrif_._	γ_denitrif._	*F*_nitrif._/*F*_denitrif._
A03	15	3.3	97.8	60.5	70.5	70.0 ± 0.9	–0.536 ± 0.004	0.774 ± 0.247	0.466 ± 0.024	4.87 ± 0.13	7.37 ± 0.82	0.737 ± 0.018	0.132 ± 0.014	0.660
A05	14	3.1	103.1	41.0	45.8	44.8 ± 0.1	–0.315 ± 0.002	0.338 ± 0.001	0.230 ± 0.008	3.24 ± 0.16	4.34 ± 0.01	0.761 ± 0.037	0.121 ± 0.001	0.748
A08	9	11.2	87.5	10.0	41.0	39.5 ± 0.2	–0.351 ± 0.007	0.118 ± 0.055	0.107 ± 0.007	2.52 ± 0.05	2.90 ± 0.18	0.689 ± 0.015	0.092 ± 0.006	0.868
A11	21	20.5	81.3	2.8	70.4	68.8 ± 1.7	–1.71 ± 0.319	0.0179 ± 0.0006	0.295 ± 0.022	2.28 ± 0.43	2.33 ± 1.04	0.291 ± 0.054	0.042 ± 0.019	0.977
F101	13	6.9	34.4	7.7	39.4	37.5 ± 0.8	–0.513 ± 0.009	0.0679 ± 0.0001	0.185 ± 0.007	2.96 ± 0.41	3.18 ± 0.03	0.632 ± 0.088	0.106 ± 0.004	0.929
F103	29	29.5	140.6	0.10	35.9	33.0 ± 0.3	–0.266 ± 0.151	(−4.70 ± 0.50) × 10^–3^	0.249 ± 0.004	3.92 ± 2.22	3.90 ± 0.49	0.820 ± 0.462	0.148 ± 0.036	1.00
F201	12	14.5	3.1	5.3	55.4	53.5 ± 0.4	–0.939 ± 0.018	0.0812 ± 0.0001	0.216 ± 0.017	2.90 ± 0.30	3.17 ± 0.06	0.488 ± 0.050	0.074 ± 0.014	0.917
P204	33	31.8	128.1		30.0		–0.337 ± 0.127		0.186 ± 0.032					
F204	33	30.8	153.1	0.27	26.3	24.1 ± 0.1	–0.224 ± 0.058	–0.0122 ± 0.0001	0.097 ± 0.011	2.92 ± 0.87	2.88 ± 0.19	0.801 ± 0.239	0.150 ± 0.020	1.01
F301	11	15.6	3.1	5.9	59.0	59.2 ± 0.9	–2.41 ± 0.399	0.107 ± 0.001	0.932 ± 0.036	0.30 ± 0.07	0.65 ± 1.30	0.037 ± 0.009	0.014 ± 0.027	0.465
F603	31	33.3	171.9	0.72	29.7	28.6 ± 0.5	–0.485 ± 0.211	–(6.80 ± 2.0) × 10^–4^	0.145 ± 0.005	1.50 ± 0.68	1.50 ± 0.69	0.489 ± 0.221	0.066 ± 0.030	1.00
J103	36	33.1	159.4	0.70	63.5	58.1 ± 0.2	–0.476 ± 0.078	–0.0370 ± 0.001	0.419 ± 0.061	7.07 ± 1.76	6.95 ± 0.26	0.821 ± 0.204	0.150 ± 0.030	1.02
P101	16	29.5	62.5	1.0	20.9	20.7 ± 0.2	–0.579 ± 0.110	(1.90 ± 0.30) × 10^–3^	0.108 ± 0.010	0.32 ± 0.07	0.33 ± 0.36	0.147 ± 0.031	0.020 ± 0.022	0.981
2A01b	41	33.7	181.3	0.37	24.4	22.5 ± 0.1	–0.080 ± 0.012	–0.0375 ± 0.0187	0.035 ± 0.002	2.35 ± 0.36	2.23 ± 0.07	0.901 ± 0.136	0.124 ± 0.004	1.05
2A02	63	33.5	181.3	0.05	14.0	12.8 ± 0.1	–0.210 ± 0.018	–0.0534 ± 0.0001	0.105 ± 0.017	1.32 ± 0.19	1.15 ± 0.06	0.660 ± 0.097	0.112 ± 0.022	1.18
2A01	33	24.6	131.3	0.80	36.4	34.0 ± 0.5	–0.556 ± 0.232	–0.0943 ± 0.0001	0.180 ± 0.019	2.73 ± 1.14	2.42 ± 0.76	0.602 ± 0.252	0.089 ± 0.028	1.12
2P03	24	34.5	50.0	0.16	47.4	45.0 ± 0.1	–0.405 ± 0.008	–0.0187 ± 0.0090	0.034 ± 0.003	3.10 ± 0.16	3.04 ± 0.04	0.703 ± 0.037	0.084 ± 0.004	1.02
2P02	16	19.8	3.1	0.16	43.8	42.3 ± 0.1	–0.665 ± 0.005	–0.0060 ± 0.0020	0.139 ± 0.009	2.03 ± 0.14	2.17 ± 0.02	0.485 ± 0.034	0.059 ± 0.001	1.01
F702	31	32.8	156.3	0.73			–0.721 ± 0.171	(3.08 ± 0.01)×10^–3^	0.260 ± 0.009					
Average (all)						40.8 ± 16.8	–0.651 ± 0.587	0.073 ± 0.205	0.251 ± 0.218	2.72 ± 1.62	2.96 ± 1.91	0.592 ± 0.245	0.093 ± 0.043	0.932 ± 0.189
Average (O_2_ > 100 μmol L^–1^)							–0.326 ± 0.164	0.012 ± 0.135				0.732 ± 0.137	0.120 ± 0.031	
Average (O_2_ < 100 μmol L^–1^)							–0.901 ± 0.700	0.127 ± 0.248				0.467 ± 0.259	0.069 ± 0.040	

a Note: All rates are in positive numbers; a negative flux number indicates efflux from sediments into the overlying waters. Bioirrigation flux and total flux of NH_4_^+^ and NO_3_^–^ across the sediment-water interface were calculated from the diffusive fluxes (see SI.2, Figure S4) and listed in Table S2. Uncertainties for calculated values (see Methods) were obtained by error propagation. The large standard deviations associated with the average values are fundamentally driven by the natural variance in organic carbon remineralization rates (indicated by SOU). Consequently, statistical comparison of unadjusted categorical means is confounded by this underlying variability. Instead, differences between the two categories are more appropriately evaluated by comparing the slopes of their respective relationships with SOU (see Figures 3 and 4 and discussion).

### Sediment N Profiles, Fluxes, and Transformations

The porewater distributions of oxygen, nitrate, and ammonium are shown in Figure 2 for selected sites along the main estuary (see Figure S1 for more locations and Figure S2 for oxygen profiles with higher vertical resolution). Nitrite was not detectable (<detection limit of 0.3 μmol L^–1^). The vertical profiles of porosity, POC, PN, and their ratios (C:N) are shown in Figure S3. The fluxes and rates of O_2_, organic C, and N species are listed in Table 1 and Table S2.

**Figure 2:**
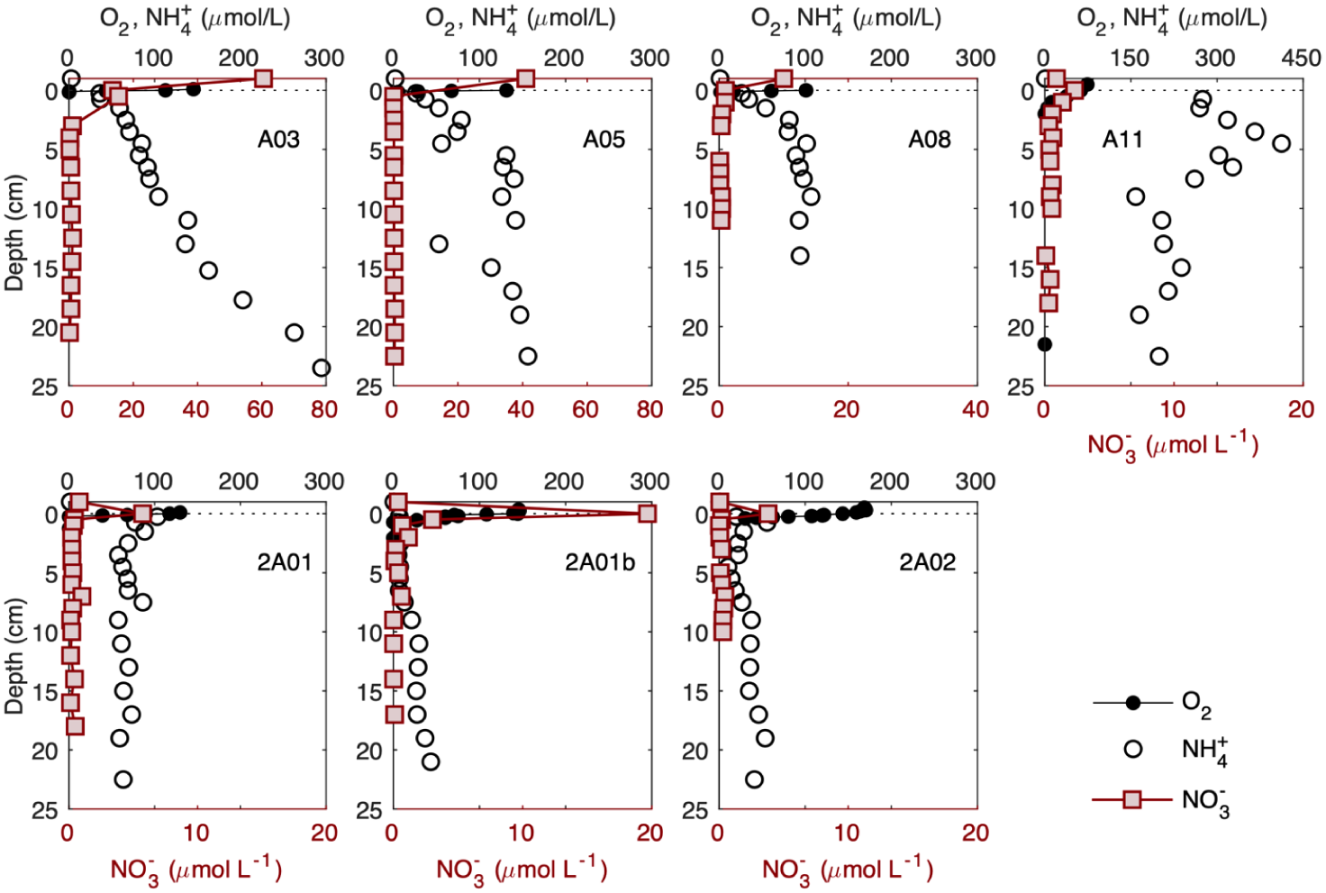
Vertical distributions of oxygen (O_2_), nitrate (NO_3_^–^), and ammonium (NH_4_^+^) in the sediments at sampling sites across the estuarine salinity gradient (see Figure S1 for profiles from other locations). Data points represent the mean of triplicate measurements. Error bars are omitted as they are smaller than the markers.

Ammonium concentration increases sharply with depth due to the degradation of organic matter in the sediments, leading to NH_4_^+^ efflux to the water column ( $F_{{\mathrm{NH}}_{4}^{+}}^{\mathrm{diff}}$ ranges from −0.080 to −2.41 mmol m^–2^ d^–1^; Table 1). However, the total NH_4_^+^ efflux, including both the molecular and bioirrigation fluxes, only corresponds to an average of 41 ± 25% of the total NH_4_^+^ produced from organic matter degradation (*F*_C_ ranges from 12.8 to 70.5 mmol m^–2^ d^–1^; Table 1), with the majority of the produced NH_4_^+^ being oxidized via nitrification within the sediments (nitrification efficiency α_nitrif._ = 59 ± 25%; Table 1). Nitrification varies strongly across the region, ranging from 4.87 ± 0.13 mmol m^–2^ d^–1^ upstream of the estuary (i.e., A03) to as low as 0.32 ± 0.07 mmol m^–2^ d^–1^ (at P101; Table 1).

Nitrification, along with the oxidation of organic matter and other reduced species, consumes oxygen, leading to the depletion of oxygen in the sediments within the top 0.3–7 mm (Figures 2 and S2). Low oxygen concentration enables anaerobic nitrate reduction, including N_2_-production processes such as denitrification and anammox,^1^ indicated by the decrease of NO_3_^–^ with depth (Figures 2 and S1). We estimate N removal (N_2_ production; $F_{N_{2}}$ ) to be in the range of 0.33–7.37 mmol m^–2^ d^–1^ (Table 1), mostly from denitrification as rates of anammox are low.^27,47^ On average, denitrification accounts for 9.3% of total organic carbon respiration (γ_denitrif._ = 9.3 ± 4.3%; Table 1).

The varying balance between denitrification and nitrification leads to strong spatial variability in the fluxes of nitrate across the sediment-water interface (Table 1). Diffusive NO_3_^–^ flux ranges from −0.037 to 0.774 mmol m^–2^ d^–1^, generally shifting from downward (into sediments) in the upper estuary (i.e., at A03, A05, and A08) to upward (efflux to the water column) offshore (e.g., at 2A01, 2A01b, 2A02, F103, and F204). In organic-rich coastal sediments within the estuary, NO_3_^–^ is rapidly consumed by intense denitrification. This high rate is driven by high organic matter loading, which supplies labile carbon substrates while its respiration consumes oxygen and promotes the anaerobic conditions required for denitrification. Consequently, the demand for NO_3_^–^ often exceeds the local supply from nitrification, particularly in the low-oxygen sediments, leading to a strong NO_3_^–^ depletion that drives a net flux of NO_3_^–^ from the water column into the sediments. In contrast, in offshore sediments with lower organic matter and thus lower denitrification rates, the NO_3_^–^ demand is lower and can often be met entirely by local nitrification, leading to excess NO_3_^–^ that fluxes out into the water column (Figures 2 and S1, Table 1).^8^

### Hypoxia Increases Sediment N Recycling and Decreases N Removal

N recycling via NH_4_^+^ efflux is generally proportional to SOU (Figure 3A), an indicator of sediment carbon mineralization (Figure S5), consistent with NH_4_^+^ production from organic matter degradation. However, the efficiency of this NH_4_^+^ recycling (the proportion released into the water column) is affected by oxygen availability in the bottom-waters. Under low bottom-water oxygen conditions, NH_4_^+^ effluxes are significantly higher (Figure 3A), with the slope of the $F_{{\mathrm{NH}}_{4}^{+}}^{\mathrm{diff}}$ vs SOU relationship – which represents the efficiency of NH_4_^+^ recycling (efflux per unit remineralization) – increasing significantly compared to high-oxygen conditions (*p* < 0.05, ANOVA). This is likely because low bottom-water oxygen level suppresses nitrification (Figure 3B), leaving more NH_4_^+^ for release into the water column. Although nitrification rates still rise with increasing SOU due to greater NH_4_^+^ availability (Figure 3B), nitrification efficiency (α_nitrif._) is significantly reduced under hypoxia (Figure 4A), evidenced by a significant flatter slope in the *F*_nitrif._ vs SOU relationship (*p* < 0.05, ANOVA; Figure 3B).

**Figure 3:**
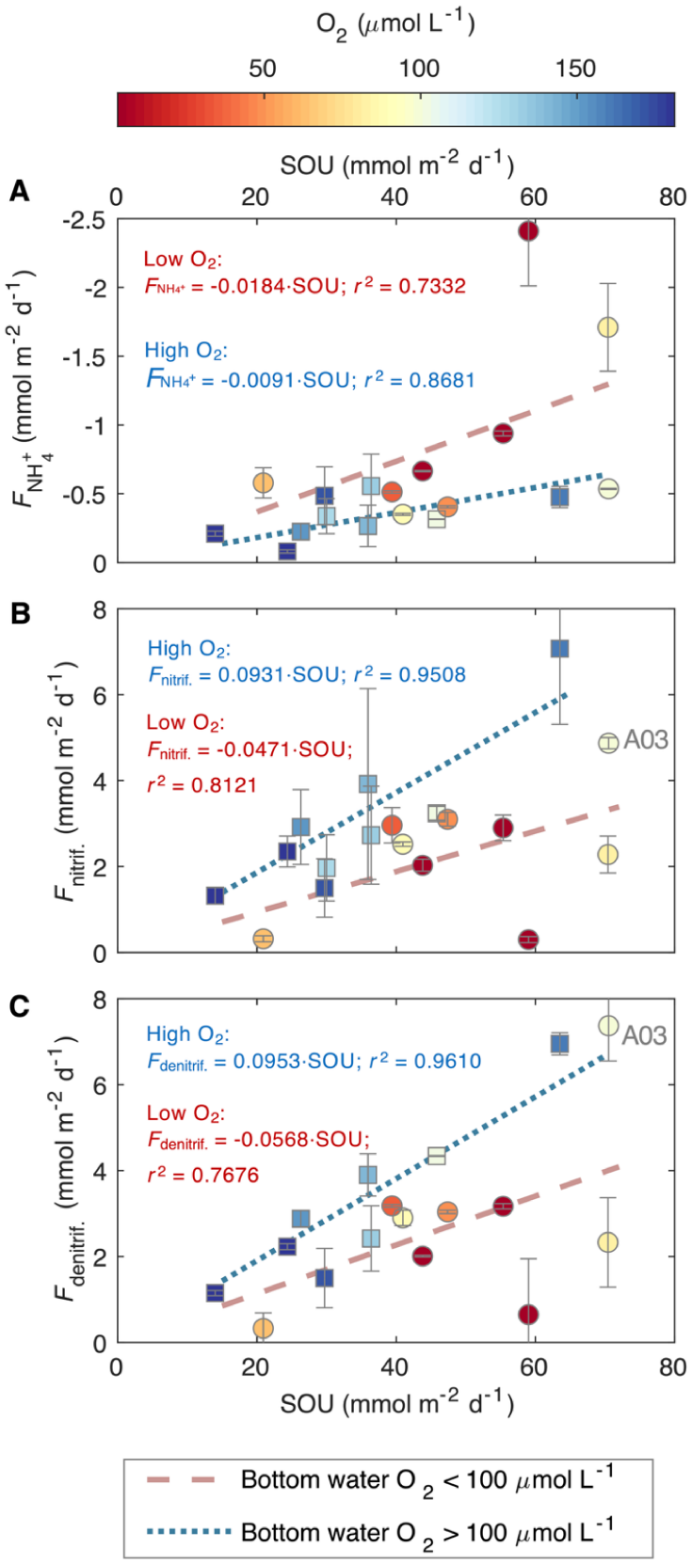
Nitrogen fluxes and rates versus sediment oxygen uptake (SOU), an estimate of the organic carbon remineralization rate: (**A**) flux of ammonium across the sediment-water interface $\left(F_{{\mathrm{NH}}_{4}^{+}}\right)$ vs SOU, (**B**) nitrification rate (*F*_nitrif._) vs SOU, and (**C**) denitrification (N_2_ production) rate (*F*_denitrif._) vs SOU. To illustrate the influence of oxygen regimes, separate linear regressions are fitted to data from the high-oxygen (O_2_ > 100 μmol L^–1^, squares) and low-oxygen (O_2_ < 100 μmol L^–1^, circles) sites. The continuous color gradient of the data markers indicates the bottom water oxygen levels. Note that the relationships likely respond progressively to oxygen decline, as shown in analyses in Figure 4A and B. Error bars were obtained by error propagation of the calculations.

**Figure 4:**
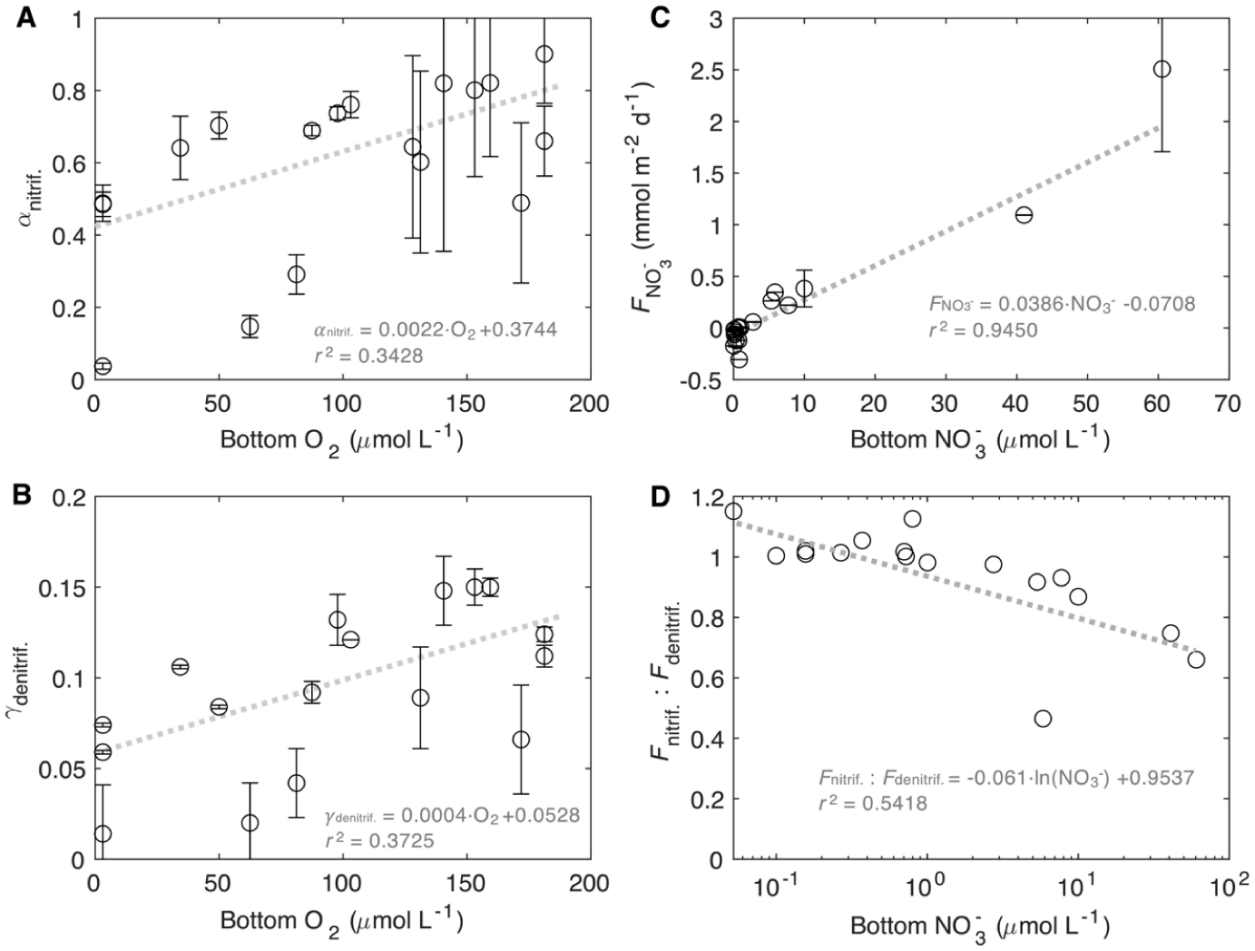
(**A**) Nitrification efficiency (α_nitrif._), defined as the proportion of remineralized ammonium that is oxidized via nitrification in the sediments, vs bottom water oxygen level; (**B**) the proportion of organic matter remineralization contributed by denitrification (γ_denitrif._) vs bottom water oxygen level; (**C**) the flux of nitrate across the sediment-water interface vs bottom water nitrate concentration and (**D**) the ratio of nitrification and denitrification rates (*F*_nitrif._:*F*_denitrif._) versus bottom water nitrate concentration. If the ratio *F*_nitrif._:*F*_denitrif._ is equal to or higher than 1, denitrification is entirely supported by nitrification. Error bars were obtained by error propagation of the calculations.

Our results suggest that the hypoxia-induced suppression of nitrification further inhibits sediment denitrification (Figure 3C), despite the latter being an anaerobic respiration process typically enhanced by low-oxygen conditions.^1,8^ At similar SOU values (organic carbon remineralization rates), the rates of N_2_ production (denitrification) are lower under reduced bottom-water oxygen concentrations (Figure 3C), and the contribution of denitrification to total organic matter remineralization declines (Figure 4B). Denitrification is supported by both organic carbon and NO_3_^–^. While the amount of organic matter is the predominant control on denitrification, as suggested by the proportional increase of denitrification and SOU (Figures 3C and S6), the supply of NO_3_^–^ also affects denitrification. NO_3_^–^ comes from either local nitrification or the overlying water. In the studied region, the flux of NO_3_^–^ into the sediments is likely limited by the low NO_3_^–^ concentrations in the bottom waters (<∼10 μmol L^–1^ at most sites, Table 1), with NO_3_^–^ flux decreasing substantially as bottom-water NO_3_^–^ level declines (Figure 4C). Consequently, denitrification heavily depends on NO_3_^–^ produced from nitrification in the surface sediments. Indeed, coupled nitrification–denitrification accounts for over 47% of the total denitrification (Table 1), with this contribution increasing as bottom-water NO_3_^–^ level declines (Figure 4D). This tight coupling is further supported by the strong correlation between the rates of sediment denitrification and nitrification rates (Figure S7), and the difference between these rates narrows when bottom-water NO_3_^–^ level is low (Figure S7B).

The effect of oxygen conditions results in contrasting sediment N budgets between high and low bottom oxygen conditions (Figure 5). Under high bottom-water oxygen conditions, about 75% of the NH_4_^+^ produced from organic matter degradation is oxidized (nitrified) in the sediments. In contrast, under low oxygen conditions, nitrification oxidizes only 45% of the produced NH_4_^+^; 55% of the remineralized NH_4_^+^ recycles back into the water column, which is significantly higher than the recycling rate observed under high-oxygen conditions (25%; *p* < 0.05, Student’s *t* test). Under high-oxygen conditions, high nitrification supports denitrification in removing 76% of the active N that is liberated into the sediments (Figure 5A). On the contrary, under low bottom O_2_ levels, suppressed nitrification restricts denitrification, reducing N removal to 53% of the total organic N remineralization (Figure 5B). Therefore, in most of the Pearl River Estuary, sediment denitrification, which is strongly dependent on nitrate supply from nitrification, is suppressed by bottom-water hypoxia, leading to reduced denitrification and overall N removal.

**Figure 5:**
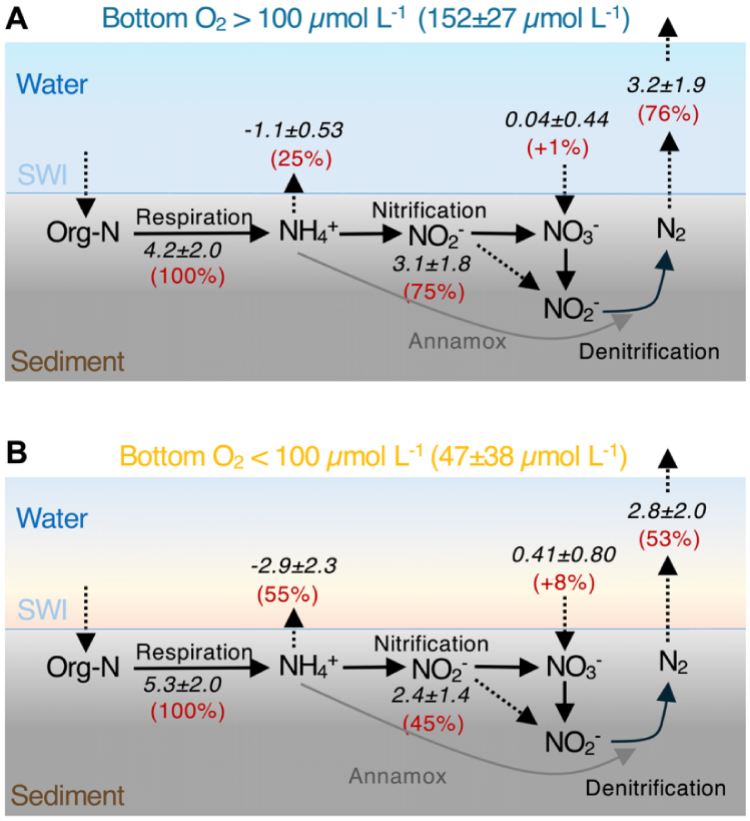
Nitrogen budgets in the sediments in the Pearl River Estuary region (**A**) under bottom water of high oxygen levels (>100 μmol L^–1^) and (**B**) under bottom water of low oxygen levels (<100 μmol L^–1^). Rates and fluxes are in the unit of mmol m^–2^ d^–1^. Average values for all sites within each group are reported, with uncertainties representing the standard deviation from the mean. The large standard deviations associated with these average values are primarily driven by natural variance in underlying organic carbon remineralization rates (indicated by SOU). Consequently, these means are not for direct statistical comparisons (see Figures 3 and 4 and the accompanying discussion for comparison of the differences between oxic and hypoxic conditions).

### Cross-System Comparisons and Broader Implications

Quantifying sediment denitrification is critical for constructing nutrient budgets and modeling ecosystem dynamics.^1,49,50^ While sedimentation removes organic nitrogen from the water column, this sequestration is only temporary. Remineralization of the sedimentary organic matter recycles this N back into the water column, predominately as ammonium (see discussions above), making it available again for primary productivity. Denitrification, critically, removes N from the system, serving as a permanent N sink.

To construct oceanic N budgets, a robust linear relationship between denitrification and SOU—a widely measured proxy of organic matter remineralization—has been established for global coastal oceans (Figure 6).^8,23,51^ This relationship indicates that denitrification accounts for a relatively stable proportion of sedimentary remineralization, effectively linking surface productivity and organic matter sedimentation to the benthic N cycle. This enables its broad application in ecosystem and biogeochemical models,^49,50^ although exceptions occur in deeply oxygenated sediments.^8^

**Figure 6:**
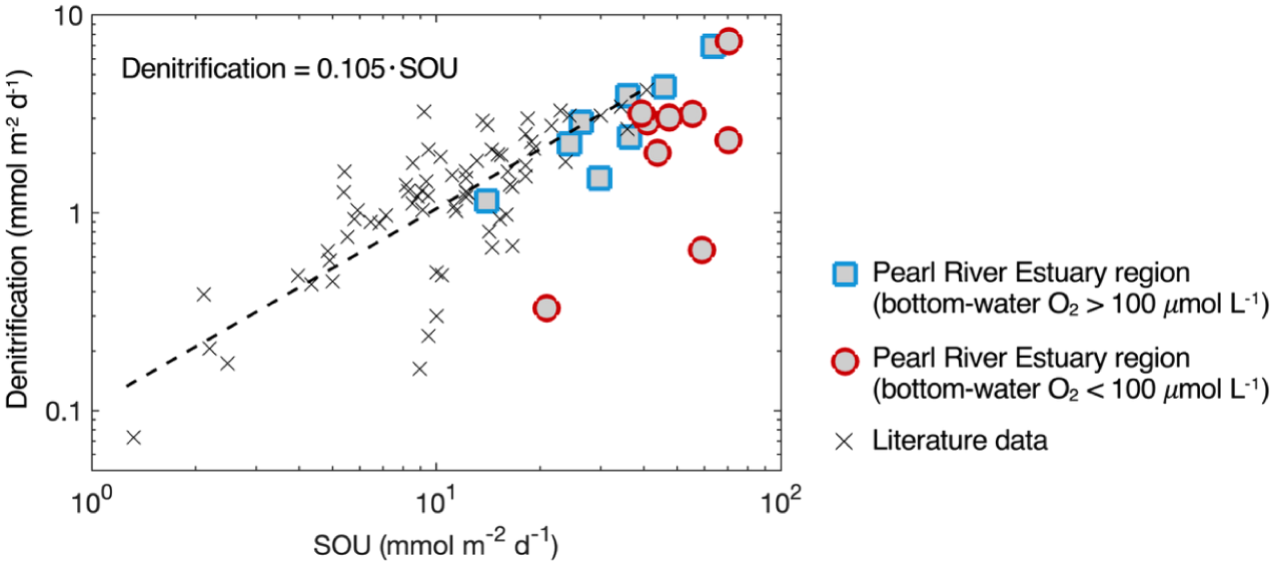
Sediment denitrification rates versus sediment oxygen uptake in continental shelf sediments. Literature data and the linear fit shown by the dashed line are from Laursen and Seitzinger (2002) and references therein.^23^

Our results for oxic sediments in the Pearl River Estuary generally align with this global trend, whereas hypoxic sites exhibit significantly lower denitrification rates (Figure 6), suggesting that the relationship requires modification under low bottom-water conditions. Consistent with our observations in a large area of the Pearl River Estuary region, low-oxygen bottom waters have been reported to promote N recycling (ammonium regeneration) and decrease sediment nitrate reduction (denitrification, anammox, and DNRA) in many other regions (Figure 7A),^14^ including the Changjiang Estuary^13^ and the Chesapeake Bay.^15,16^ Conversely, other studies have shown that certain systems can sustain a high level of denitrification despite suppressed nitrification.^9,11,12^ This is similar to the upper estuary within the Pearl River Estuary region (A03), which is likely due to the relatively higher supply of NO_3_^–^ from the water column to support denitrification, evidenced by the substantially higher NO_3_^–^ influx compared to other sites (Table 1).

**Figure 7:**
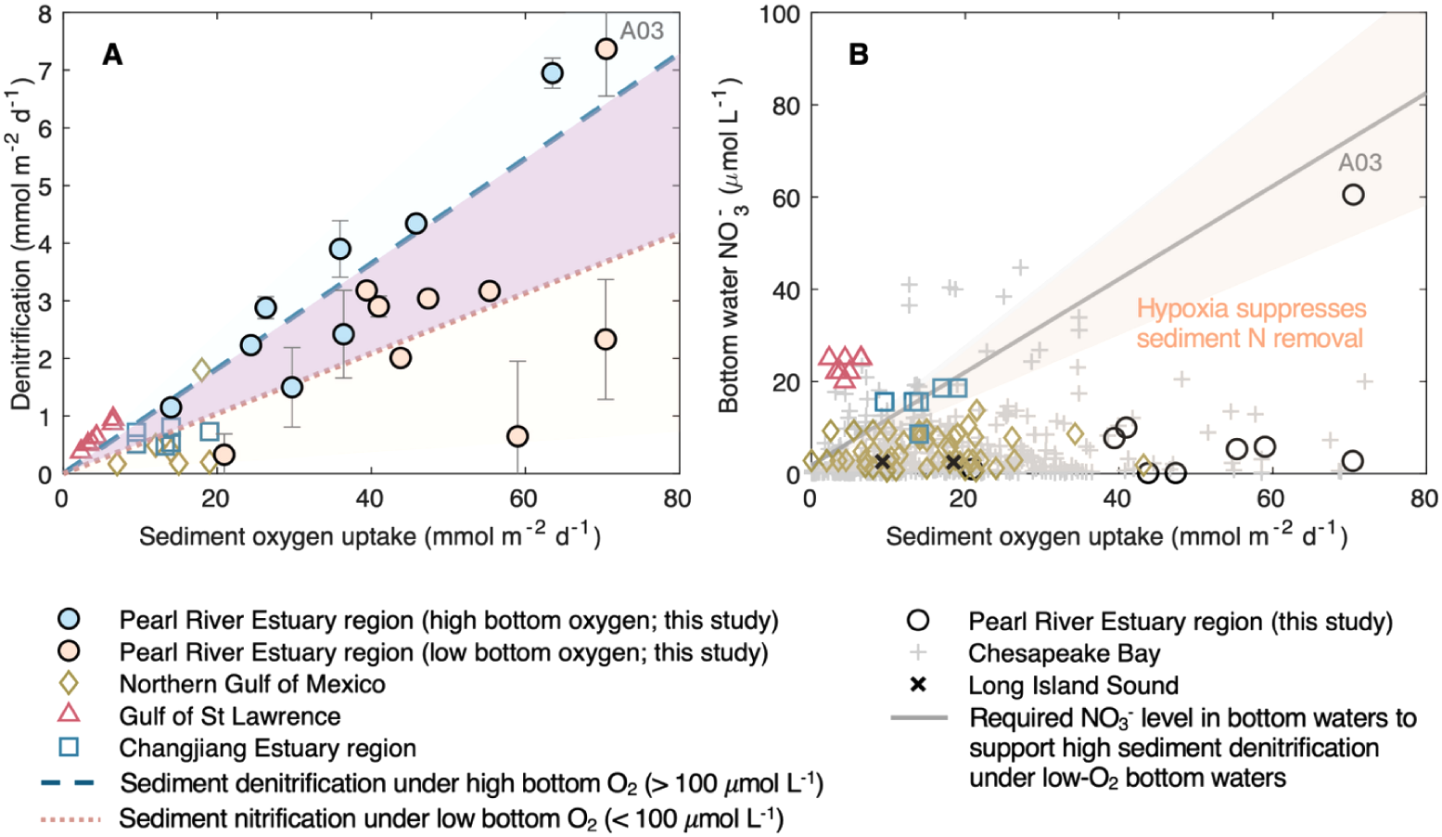
Comparison of coastal ocean sediments: (**A**) Denitrification rate vs sediment oxygen uptake (SOU) and (**B**) bottom water nitrate concentration vs SOU for sediments under low-oxygen bottom waters. Data include those of the Pearl River Estuary region (this study), the northern Gulf of Mexico,^9,59,60^ the Gulf of St Lawrence,^61,62^ the Changjiang Estuary region,^13^ the Long Island Sound,^63^ and the Chesapeake Bay^5,64,65^ (see SI.5 and Table S3). In panel (A), sediments under high (O_2_ > 100 μmol L^–1^) and low bottom-oxygen conditions (O_2_ < 100 μmol L^–1^) are indicated using markers filled with light blue and pink colors, respectively. The dashed blue line represents the calculated theoretical rate of denitrification under high bottom-oxygen conditions, with the lighter blue shaded area indicating the upper bound of uncertainty; the dotted pink line indicates the calculated rate of nitrification under low bottom-oxygen conditions, with the lighter pink shaded area indicating the lower bound of uncertainty. The difference between the dashed blue line and the dotted pink line (the purple area) indicates the flux of NO_3_^–^ required from the bottom waters to support an unsuppressed rate of denitrification under low bottom oxygen. This difference was used to calculate the concentration of nitrate in the bottom waters needed to sustain an unsuppressed rate of denitrification, shown in panel (B) as the gray line, with the uncertainty shown as the gray shaded area. In systems below this threshold value (within the pink shaded area), hypoxia decreases sediment N removal.

We can use a mass balance analysis to understand this variability. The expected rate of denitrification under high bottom-oxygen conditions can be calculated for sediments of corresponding SOU using an average remineralization stoichiometry of *r*_C:N_ = 8.5 ± 1.5 (Table S2), an average proportion of NH_4_^+^ that is oxidized in the sediments (α_nitrif._ = 0.732 ± 0.127; Table 1), and the average proportion of organic matter remineralization attributed to denitrification (γ_denitrif._ = 0.120 ± 0.031; Table 1) under high-oxygen conditions. This hypothetical denitrification rate is plotted in Figure 7A (the dashed blue line) in comparison with the observations in the Pearl River Estuary Region and other coastal systems. Under high bottom-water oxygen conditions, nitrification is consistently sufficient. As organic matter sedimentation increases, it releases more NH_4_^+^, which enhances nitrification and thereby supporting a proportionally increase in denitrification (Figure 7A). As organic matter sedimentation (and remineralization indicated by SOU) increases further, which often triggers hypoxia, nitrification can be limited despite a high production of NH_4_^+^ from organic matter remineralization. This suppressed rate of nitrification can be calculated (from eq 11) using an average α_nitrif._ of 0.467 ± 0.259 under low bottom-water oxygen conditions (Table 1), as shown in Figure 7A as the dotted red line. Under such limited nitrification, the additional nitrate flux is required from the overlying waters to support a “normal” unsuppressed activity of denitrification, and this flux can be calculated as the difference between the “theoretical” rates of denitrification (under high-O_2_ conditions; dashed blue line) and suppressed hypoxic nitrification rates (dotted red line). This difference, illustrated as the purple area in Figure 7A, increases with increasing SOU because systems with higher rates of organic matter remineralization would require greater nitrate fluxes to maintain the unsuppressed rate of denitrification under suppressed nitrification.

The flux of nitrate into sediments is strongly limited by its concentration in the overlying waters, though other sedimentary factors also play a role.^52,53^ Using the empirical relationship between nitrate flux and bottom-water nitrate concentration (Figure 4C), we can then roughly estimate the required level of nitrate in the bottom water to support an unsuppressed activity of denitrification under hypoxia-suppressed nitrification (the purple area in Figure 7A). The theoretical values are plotted in Figure 7B as the gray line, compared with observed bottom-water nitrate levels for the hypoxia sites in the Pearl River Estuary region and other similar systems. At locations where bottom-water nitrate concentrations fall below the required threshold (the shaded area in Figure 7B), the nitrate flux from the bottom water is insufficient to compensate for the reduced nitrate supply from nitrification, leading to decreased denitrification. This explains the observations in the Pearl River Estuary region (except at A03), where bottom-water nitrate levels are mostly below the threshold value (Figure 7B), leading to decreased N removal under hypoxia (Figure 7A). On the contrary, at site A03, bottom-water nitrate is high and close to the threshold value (Figure 7B), consistent with the high denitrification rate there (Figure 7A). Similarly, the Gulf of St Lawrence has higher bottom-water nitrate concentrations compared to the threshold nitrate levels (Figure 7B), explaining the relatively high rates of sediment denitrification even under low bottom-water oxygen conditions (Figure 7A). On the contrary, the northern Gulf of Mexico exhibit substantial reduction of sediment denitrification under low bottom-water oxygen levels (Figure 7A), consistent with their low bottom-water nitrate concentrations (Figure 7B). The Long Island Sound and the Chesapeake Bay – two major hypoxic systems in the world’s coastal oceans^54^ – mostly fall within the low-nitrate category, where bottom-water hypoxia likely decreases sediment N removal (Figure 7B).

Several caveats of the analysis warrant consideration. The nitrate and ammonium fluxes were estimated considering proportional bioirrigation and molecular diffusion coefficients^43^ (see Methods), but the actual fluxes should lie between purely diffusive fluxes ( $F_{{\mathrm{NH}}_{4}^{+}}^{\mathrm{diff}}$ and $F_{{\mathrm{NO}}_{3}^{-}}^{\mathrm{diff}}$ ) and the estimated totals (see SI.2). Nevertheless, even in the no-bioirrigation scenario (diffusive flux only), the effects of bottom-water hypoxia on sediment nitrification and denitrification rates remain significant (Figure S6; *p* < 0.05 for both *F*_nitrif._ vs SOU and *F*_denitrif._ vs SOU, ANOVA), supporting our conclusion that low bottom-water oxygen suppresses nitrification and reduces sediment N removal.

Potential variabilities in reaction stoichiometries should also be considered. For examples, the incomplete ammonium oxidation to NO_2_^–^, followed by its denitrification, follows different stoichiometries than our model assumes. However, results from a modified model accounting for this variability (SI.4) indicate the results are insensitive to these changes (Figure S8; SI.4). The production of N_2_O from incomplete denitrification was not considered. While the resulting error is likely small, given the minor stoichiometric difference between complete (4C: 5NO_3_^–^) and incomplete (1C:1NO_3_^–^) denitrification, this process warrants further investigation as N_2_O is a potent greenhouse gas.

Sediment N removal via anammox was not quantified in our study due to its minor contribution in the region.^27,47^ However, anammox is globally significant.^13,55,56^ The observed hypoxia-driven suppression of nitrification, which limits denitrification, should similarly limit anammox, as it also depends on nitrate supply. This further reduces overall sediment N removal under bottom-water hypoxia.

The use of SOU as a proxy for organic carbon mineralization, while common,^8,20,23,40,57,58^ may introduce uncertainties in our analysis using literature data. Under bottom-water hypoxia, SOU is limited by low oxygen and underestimates sediment organic matter remineralization.^38^ We address this issue in this study by measuring SOU under sufficient oxygen (see Methods and SI.1), but this has not been commonly conducted in previous studies (e.g., literature data in Figures 6 and 7).^59^ If we account for the potential underestimation of organic carbon remineralization in the literature, some systems (locations) currently above the threshold nitrate value would fall into the low-nitrate category (Figure 7B), meaning that more sediments (e.g., from the northern Gulf of Mexico and the Chesapeake Bay) would likely lack sufficient bottom-water nitrate to maintain normal denitrification rates under hypoxia (Figure 7B). This suggests that increasing coastal hypoxia likely reduces sediment N removal more broadly. This suppression of denitrification increases recycling of inorganic N to support water-column primary productivity, thereby creating an amplifying feedback loop that exacerbates coastal eutrophication and hypoxia, particularly in N-limiting systems.

## Supplementary Material



## Data Availability

Data presented in the paper are openly available at DataSpace@HKUST via 10.14711/dataset/XMTKWW.

## Notes

Measurement of oxygen sediment uptake to estimate organic carbon mineralization rate; estimation of bioirrigation flux of ammonium and nitrate; calculation of ammonium burial flux in the sediments; the mass-balance equations considering the variability in reaction stoichiometry; data sources from literature and the treatment of the data; a table (Table S1) summarizing reactions in sediments associated with nitrogen cycling, and two tables (Tables S2 and S3) including additional data from the studied region and literature; additional figures supporting the discussion, including sediment profiles of oxygen, nitrate, and ammonium for other sites that are not presented in the main text (Figure S1), oxygen profiles with a higher vertical resolution (Figure S2), vertical profiles of sediment porosity, particulate organic carbon, particulate nitrogen, and the ratios of C:N (Figure S3), sediment total oxygen uptake vs diffusive oxygen uptake (Figure S4), organic carbon remineralization vs sediment oxygen uptake (Figure S5), sediment nitrification and denitrification rates estimated using only molecular diffusion fluxes (Figure S6), the relationships between sediment denitrification and nitrification rates (Figure S7), and rates organic carbon remineralization, nitrification, and denitrification estimated using models with incomplete nitrification vs complete nitrification (Figure S8) (PDF)
