# Supplementary material for: Hypoxia Decreases Nitrogen Removal in Coastal Marine Sediments
Source: Environ Sci Technol. 2026 Mar 30;60(14):10763–74. doi: 10.1021/acs.est.5c04877 (PMC13085807; doi:10.1021/acs.est.5c04877)
Supplement: Supplementary file 1 [file es5c04877_si_001.pdf]

Supporting Information for

Hypoxia decreases nitrogen removal in coastal marine sediments

Jing Sun[1], Xingyu Yang[1], Liuqian Yu[2,3], Qiong Zhang[1,2], Charmaine C. M. Yung[1], and Jiying Li[1,2*]

[1]Department of Ocean Science, The Hong Kong University of Science and Technology, Clear Water Bay, Kowloon, Hong Kong SAR, P. R. China

[2]Center for Ocean Research in Hong Kong and Macau, Hong Kong SAR, P. R. China

[3]Earth, Ocean and Atmospheric Sciences Thrust, The Hong Kong University of Science and Technology (Guangzhou), Guangdong 511400, P.R. China

* Corresponding author: email: jiyingli@ust.hk; tel.: +852 3469 2934

This supplementary document spans 21 pages, containing five text sections (SI.1-SI.5), three tables (Tables S1-S5), and seven figures (Figs. S1-S7):

SI.1 Measurement of sediment oxygen uptake to estimate organic carbon remineralization rate

SI.2 Estimation of bioirrigation flux of ammonium and nitrate

SI.3 Calculation of ammonium burial flux in the sediments

SI.4 The mass-balance equations considering the variability in reaction stoichiometry

SI.5 Data sources from the literature and the treatment of the data

Table S1 Reactions in sediments associated with nitrogen cycling.

Table S2 Sampling location, total water depth, the ratio of organic carbon and nitrogen remineralization in surface sediments ($r_{C:N}$), sediment oxygen diffusive flux across the sediment-water interface (SWI) ($F_{O_2}^{diff}$), bioirrigation fluxes of ammonium and nitrate across the SWI ($F_{NH_4^+}^{irr}$ and $F_{NO_3^-}^{irr}$), and total fluxes of ammonium and nitrate ($F_{NH_4^+}$ and $F_{NO_3^-}$) across the SWI.

Table S3 Data for other coastal oceans under seasonal hypoxia.

Figure S1 Vertical distributions of oxygen, nitrate, and ammonium in the sediments.

Figure S2 Vertical distributions of oxygen in the sediments

Figure S3 Vertical profiles of sediment porosity, particulate organic carbon (POC), particulate nitrogen (PN), and the ratios of POC and PN (C:N).

Figure S4 Sediment oxygen uptake (total) vs diffusive oxygen uptake

Figure S5 The rates of organic carbon remineralization in sediments versus sediment oxygen uptake.

Figure S6 Sediment nitrification and denitrification rates versus sediment oxygen uptake in the Pearl River Estuary region. The rates were estimated using molecular diffusive fluxes of nitrate and ammonium.

Figure S7 (**A**) Sediment denitrification rate vs nitrification rate, (**B**) the difference between nitrification and denitrification rates vs bottom water nitrate concentration, and (**C**) the proportion of organic matter remineralization contributed by denitrification ($\gamma_{\text{denitrif.}}$) vs the proportion of remineralized ammonium that is oxidized via nitrification in the sediments ($\alpha_{\text{nitrif.}}$)

Figure S8 Rates organic carbon remineralization, nitrification, and denitrification estimated using models with incomplete nitrification vs complete nitrification.

SI.1 Measurement of sediment oxygen uptake (SOU) to estimate organic carbon remineralization rate

Sediment oxygen uptake (SOU), defined as the downward flux of oxygen into the sediment across the sediment-water interface, was determined using whole-core incubations. Intact sediment cores with approximately 15-20 cm of overlying waters were stabilized, and the overlying water was gently bubbled with air to compensate for oxygen lost between sample collection and incubation. To perform the incubation, sediment cores were tightly sealed, and the overlying waters were gently stirred with a hanging stir bar located at ~5 cm above the sediment-water interface. Oxygen concentrations in the overlying water was monitored using an oxygen optical sensor (OXROB10, Pyroscience GmbH) compensated using the experimental temperature measured by a temperature sensor (TDIP15, Pyroscience GmbH). The incubation was conducted under room temperature of 23-26 °C, which is similar to the in-situ bottom water temperature. The incubation typically lasted around 2-3 hours and SOU (mmol O_2 m^{-2} d^{-1}) was obtained from the linear decrease of oxygen in the overlying water. The methods and results of SOU are reported in our previous study[1].

SOU is commonly measured to approximate the rate of organic carbon mineralization in sediments, because oxygen is consumed either directly in the oxidation of organic carbon or in the oxidation of the reduced species (e.g., Fe^{2+}, Mn^{2+}, H_2S) produced by anaerobic carbon degradation. This is evidenced by the low concentrations of these reduced species (Fe^{2+}, Mn^{2+}, H_2S) in the surface sediments and the overlying waters (data not shown). As a result, oxygen serves as the ultimate electron acceptor for organic carbon, and a stoichiometry close to 1C:1O_2 stoichiometry is maintained. An exception is N_2 produced via denitrification, which does not consume oxygen. Therefore, denitrification is corrected when relating SOU to the rate of organic carbon remineralization (see Eq. 7 in the main text):

$$\mathrm{SOU} = \mathrm{SOU_C} + \mathrm{SOU_{nitrif.}} = (1-\gamma_{\mathrm{denitrif.}})F_\mathrm{C} + \frac{2}{r_{\mathrm{C:N}}}\alpha_{\mathrm{nitrif.}}F_\mathrm{C} \quad \text{Eq. S1}$$

Here, SOU_C and $SOU_{nitrif.}$ are SOU due to carbon remineralization (1C:1O_2) and nitrification (1NH_4^+:2O_2), respectively (Table S1); $\gamma_{denitrif.}$ is the proportion of organic matter remineralization contributed by denitrification; $\alpha_{nitrif.}$ is the proportion of NH_4^+ being nitrified in the total amount of NH_4^+ remineralized from organic matter in the sediments. F_C is the rate of organic carbon remineralization (all rates are positive numbers hereafter) following a remineralization C:N ratio ($r_{C:N}$).

However, in sediments under low bottom-water oxygen concentrations (< 1.5-3.2 mg L^{-1}), SOU can be limited by oxygen availability[2], leading to underestimation of organic carbon mineralization rates using SOU (Eq. 7 in the main text), because some reduced species might be released into the water column. To address this potential issue at the hypoxia sites, where sediment cores collected under hypoxic bottom waters (O_2 < 62.5 μmol L^{-1}), we bubbled sufficient air into the overlying waters before the incubation to ensure that oxygen uptake was not limited by low oxygen concentration. In other words, anaerobic respiration (anaerobic oxidation of organic matter) is accounted for because oxygen will be used to oxidize the produced reduced species. This approach overestimates in-situ SOU at these low-oxygen sites, but it provides a more accurate estimate of organic matter remineralization, better serving the purpose of this study and Eq. S1 (Eq.7 in the main text) remains valid.

SI.2 Estimation of bioirrigation flux of ammonium and nitrate

The transport of solutes by bioirrigation can be considered as enhanced diffusivity analogous to eddy diffusion[3], because such enhancement is introduced by the diffusion of molecules between overlying water filled into the burrows and the porewater around the burrows. Therefore, bioirrigation diffusion is typically described as being proportional to the concentration difference between the porewater and overlying waters (water in the burrows) corrected by bioirrigation coefficients[4,5]:

$$F_i^{\text{irr}} = \alpha_i^{\text{irr}}(C_i^{\text{aver}} - C_i^0)L_{\text{irr}} \quad \text{Eq. S2}$$

where F_i^{irr} is the bioirrigated flux; α_i^{irr} is the bioirrigation coefficient; C_i^{aver} and C_i^0 are the concentrations of the species of interest in the porewater of the bioturbated zone and the overlying waters (burrows), respectively. L_{irr} is the depth of bioirrigation. Bioirrigation coefficients are proportional to molecular diffusion coefficients (D_i^{s})[3]:

$$\frac{\alpha_i^{\text{irr}}}{\alpha_j^{\text{irr}}} = \frac{D_i^{\text{s}}}{D_{\text{j}}^{\text{s}}} \quad \text{Eq. S3}$$

Hence, we can estimate the bioirrigated flux of other species using those of oxygen:

$$\frac{F_i^{\text{irr}}}{F_{\text{O}_2}^{\text{irr}}} = \frac{\alpha_i^{\text{irr}}(C_i^{\text{aver}} - C_i^0)L_{\text{irr}}}{\alpha_{\text{O}_2}^{\text{irr}}(C_{\text{O}_2}^{\text{aver}} - C_{\text{O}_2}^0)L_{\text{irr}}} = \frac{D_i^{\text{s}}(C_i^{\text{aver}} - C_i^0)}{D_{\text{O}_2}^{\text{s}}(C_{\text{O}_2}^{\text{aver}} - C_{\text{O}_2}^0)} \quad \text{Eq. S4}$$

Since the average porewater concentration around the burrows (C_i^{aver}) is not known (depending on the length of the burrow), we cannot calculate its difference from the concentration in the overlying waters ($C_i^{\text{aver}} - C_i^0$). We then approximate Eq. S4 using the concentration gradient across the sediment-water interface:

$$\frac{F_i^{\text{irr}}}{F_{\text{O}_2}^{\text{irr}}} = \frac{D_i^{\text{s}}(C_i^{\text{aver}} - C_i^0)}{D_{\text{O}_2}^{\text{s}}(C_{\text{O}_2}^{\text{aver}} - C_{\text{O}_2}^0)} = \frac{D_i^{\text{s}}(C_i^{\Delta x} - C_i^0)/\Delta x}{D_{\text{O}_2}^{\text{s}}(C_{\text{O}_2}^{\Delta x} - C_{\text{O}_2}^0)/\Delta x} = \frac{F_i^{\text{diff}}}{F_{\text{O}_2}^{\text{diff}}} \quad \text{Eq. S5}$$

where $C_i^{\Delta x}$ is the concentration at Δx, and F_i^{diff} is diffusive flux. This approximation is based on the understanding that a steeper concentration gradient across the sediment-water interface would lead to a larger difference between the concentrations in overlying water and in the porewaters around the burrows in the shallow sediments. Therefore, the bioirrigated flux of species *i* can be estimated as

$$F_i^{\text{irr}} = \frac{F_i^{\text{diff}}}{F_{\text{O}_2}^{\text{diff}}} F_{\text{O}_2}^{\text{irr}} \quad \text{Eq. S6}$$

The bioirrigated flux of oxygen ($F_{\text{O}_2}^{\text{irr}}$) can be approximated by the difference between the SOU measured by incubation and the sediment oxygen diffusive flux ($F_{\text{O}_2}^{\text{diff}}$):

$$F_{O_2}^{irr} = SOU - F_{O_2}^{diff} \qquad \text{Eq. S7}$$

Diffusive flux can be estimated using Fick's law of diffusion across the sediment-water interface:

$$F_i^{diff} = -\varphi D_i^s \frac{dC_i}{dz} \qquad \text{Eq. S8}$$

To estimate the bioirrigation flux of of NO_3^- and NH_4^+, a correction factor was first obtained the relationship between SOU and $F_{O_2}^{diff}$ (Fig. S3): SOU = 3.24 $F_{O_2}^{diff}$, which indicates that the bioirrigation flux for oxygen is 224% of its molecular diffusive flux. This translates to the following corrections for bioirrigation fluxes of NO_3^- and NH_4^+:

$$F_i^{irr} = 224\% F_i^{diff} \qquad \text{Eq. S7}$$

This approximation carries some uncertainties. First, the approximation in Eq. S5 assumes that changes in concentration with depth within the bioirrigation zone follow a linear fashion, which may be true if the bioirrigation zone (average depth of the burrows) is very thin. However, bioirrigation depth is likely to be deeper than the linear range. Moreover, the diffusive oxygen uptake ($F_{O_2}^{diff}$) is likely underestimated using Eq. S8 because the vertical measurement resolution (0.5 mm) is coarser than the theoretical infinitesimal scale needed to define the concentration gradient across the sediment-water interface. The actual total fluxes of nitrate and ammonium should fall between their molecular diffusive fluxes ($F_{NH_4^+}^{diff}$ and $F_{NO_3^-}^{diff}$) and estimated total fluxes.

SI. 3 Calculation of ammonium burial flux in the sediments

The adsorption of ammonium (NH_4^+) by sediment particles can be described using a linear adsorption model[6]:

$$K_{\mathrm{NH4}} = \frac{C_{\mathrm{NH_4^+}}^{\mathrm{sorbed}}}{C_{\mathrm{NH4}}} \qquad \text{Eq. S1}$$

where K_{NH4} is the unitless adsorption coefficient of NH_4^+ in sediments, measured to be around 1.3 for coastal surface marine sediments of porosity less than 0.9[6]; $C_{\mathrm{NH_4^+}}^{\mathrm{sorbed}}$ is the amount of NH_4^+ adsorbed per unit volume of porewater in the sediments; $C_{NH_4^+}$ is the NH_4^+ concentration in the porewater (e.g., in μmol L^{-1}). The sediment burial flux of adsorbed NH_4^+ ($F_{\mathrm{NH_4^+}}^{\mathrm{bur}}$) is

$$F_{\mathrm{NH_4^+}}^{\mathrm{bur}} = C_{\mathrm{NH_4^+}}^{\mathrm{sorbed}} \frac{\varphi}{(1-\varphi)} \frac{1}{\rho} F_{\mathrm{sed}}^{\mathrm{bur}} = C_{\mathrm{NH4}} K_{\mathrm{NH4}} \frac{\varphi}{(1-\varphi)} \frac{1}{\rho} F_{\mathrm{sed}}^{\mathrm{bur}} \qquad \text{Eq. S2}$$

Here, φ is the sediment porosity; ρ is the density of dry sediments, and $F_{\mathrm{sed}}^{\mathrm{bur}}$ is the sediment burial rate (e.g., in g cm^{-2} y^{-1}). For example, for a sediment burial rate of $F_{\mathrm{sed}}^{\mathrm{bur}}$ = 1 g cm^2 y^{-1}, an NH_4^+ concentration of $C_{NH_4^+}$ = 500 μmol L^{-1}, sediment porosity of φ= 0.5, and dry density of ρ = 2.3 g cm^{-3}, NH_4^+ burial flux is calculated (Eq. S2) to be around 0.007 mmol m^{-2} d^{-1}. This ammonium burial flux is an overestimate because in the sediments of the Pearl River Estuary region, the sediment burial rate is generally < 1 g cm^2 y^{-1} [7,8]; ammonium concentrations in the sediments are generally lower than 500 μmol L^{-1} (Figs. 2 and S1); porosity in the sediments below 20 cm is in the range of 0.5–0.8 and sediment dry density is ~2.5 g cm^{-3} [9]. Such an overestimate is still 1–2 orders of magnitude lower than the efflux of NH_4^+ from the sediments into the water column (see Tables 1 and S2). Therefore, we consider the NH_4^+ burial flux negligible in the sediment nitrogen budget.

SI.4 The mass-balance equations considering the variability in reaction stoichiometry

The following mass-balance equations describe the sediment N budget, including

1) mass balance for NH_4^+:

$$F_{\mathrm{NH_4^+}} = -\left(\left(1 - \alpha_{\mathrm{nitrif.}}\right) \frac{F_{\mathrm{C}}}{r_{\mathrm{C:N}}} - F_{\mathrm{NH_4^+}}^{\mathrm{bur}}\right) \qquad \text{Eq. S1}$$

Here, $\alpha_{\text{nitrif.}}$ is the proportion of NH_4^+ being nitrified in the total amount of NH_4^+ remineralized from organic matter in the sediments. F_C is the rate of organic carbon remineralization (all rates are positive numbers hereafter) following a remineralization C:N ratio ($r_{\text{C:N}}$). $F_{\text{NH}_4^+}^{\text{bur}}$ is the burial flux of NH_4^+ in the sediments.

2) mass balance for O_2:

$$\text{SOU} = \text{SOU}_\text{C} + \text{SOU}_{\text{nitrif.}} = (1 - \gamma_{\text{denitrif.}})F_\text{C} + \frac{2}{r_{\text{C:N}}}\alpha_{\text{nitrif.}}F_\text{C} \qquad \text{Eq. S2}$$

SOU_C and $\text{SOU}_{\text{nitrif.}}$ are SOU due to carbon remineralization (1C:1O_2) and nitrification (1NH_4^+:2O_2), respectively (Table S1; see later for stoichiometry variability); $\gamma_{\text{denitrif.}}$ is the proportion of organic matter remineralization contributed by denitrification.

3) C and N balance for denitrification:

$$F_{\text{denitrif.}} = \frac{4}{5}\gamma_{\text{denitrif.}}F_\text{C} \qquad \text{Eq. S3}$$

and 4) mass balance for inorganic N:

$$\frac{1}{r_{\text{C:N}}}F_\text{C} + F_{\text{NO}_3^-} + F_{\text{NH}_4^+} - F_{\text{NH}_4^+}^{\text{bur}} - F_{\text{denitrif.}}$$

$$= \frac{1}{r_{\text{C:N}}}F_\text{C} + F_{\text{NO}_3^-} + F_{\text{NH}_4^+} - F_{\text{NH}_4^+}^{\text{bur}} - \frac{4}{5}\gamma_{\text{denitrif.}}F_\text{C} = 0 \qquad \text{Eq. S4}$$

The solutions of Eqs. S1-4 for the three unknown variables F_C, $\alpha_{\text{nitrif.}}$ and $\gamma_{\text{denitrif.}}$ are:

$$F_\text{C} = \frac{\left(\text{SOU} + \frac{5}{4}F_{\text{NO}_3^-} - \frac{3}{4}\left(F_{\text{NH}_4^+} - F_{\text{NH}_4^+}^{\text{bur}}\right)\right)}{\left(1 + \frac{3}{4r_{\text{C:N}}}\right)} \qquad \text{Eq. S5}$$

$$\gamma_{\text{denitrif.}} = \frac{5}{4F_\text{C}}\left(\frac{F_\text{C}}{r_{\text{C:N}}} + F_{\text{NO}_3^-} + F_{\text{NH}_4^+} - F_{\text{NH}_4^+}^{\text{bur}}\right) \qquad \text{Eq. S6}$$

$$\alpha_{\text{nitrif.}} = \left(F_{\text{NH}_4^+} - F_{\text{NH}_4^+}^{\text{bur}}\right)\frac{r_{\text{C:N}}}{F_\text{C}} + 1 \qquad \text{Eq. S7}$$

The rates of denitrification can be calculated using Eq. S3, and nitrification can be calculated as:

$$F_{\text{nitrif.}} = \frac{1}{r_{\text{C:N}}}\alpha_{\text{nitrif.}}F_\text{C} \qquad \text{Eq. S8}$$

For an **incomplete nitrification** that produces NO_2^- instead of NO_3^- ($1NH_4^+:1.5O_2$), Eq. S2 becomes:

$$\mathrm{SOU} = \mathrm{SOU_C} + \mathrm{SOU_{nitrif.}} = (1-\gamma_{\mathrm{denitrif.}})F_{\mathrm{C}} + \frac{3}{2r_{\mathrm{C:N}}}\alpha_{\mathrm{nitrif.}}F_{\mathrm{C}} \qquad \text{Eq. S9}$$

The produced NO_2^- further undergoes denitrification (Table S1) and Eq. S3 becomes:

$$F_{\mathrm{denitr.}} = \frac{4}{3}\gamma_{\mathrm{denitrif.}}F_{\mathrm{C}} \qquad \text{Eq. S10}$$

and Eq. S4 becomes

$$\frac{1}{r_{\mathrm{C:N}}}F_{\mathrm{C}} + F_{\mathrm{NO_3^-}} + F_{\mathrm{NH_4^+}} - F_{\mathrm{NH_4^+}}^{\mathrm{bur}} - F_{\mathrm{denitrif.}} = 0 \qquad \text{Eq. S11}$$

Therefore, the solutions for Eqs. S1, S8, S9, and S10 are:

$$F_{\mathrm{C}} = \frac{\left(\mathrm{SOU}+\frac{3}{4}F_{\mathrm{NO_3^-}}-\frac{3}{4}\left(F_{\mathrm{NH_4^+}}-F_{\mathrm{NH_4^+}}^{\mathrm{bur}}\right)\right)}{\left(1+\frac{3}{4r_{\mathrm{C:N}}}\right)} \qquad \text{Eq. S12}$$

$$\gamma_{\mathrm{denitrif.}} = \frac{3}{4F_{\mathrm{C}}}\left(\frac{F_{\mathrm{C}}}{r_{\mathrm{C:N}}} + F_{\mathrm{NO_3^-}} + F_{\mathrm{NH_4^+}} - F_{\mathrm{NH_4^+}}^{\mathrm{bur}}\right) \qquad \text{Eq. S13}$$

$$\alpha_{\mathrm{nitrif.}} = \left(F_{\mathrm{NH_4^+}} - F_{\mathrm{NH_4^+}}^{\mathrm{bur}}\right)\frac{r_{\mathrm{C:N}}}{F_{\mathrm{C}}} + 1 \qquad \text{Eq. S14 (same as Eq. S7)}$$

We can then estimate denitrification using Eq. S10 and nitrification using Eq. S8. These estimates the rates under the extreme scenario that nitrification follows only the "incomplete" pathway (Table S1).

The results obtained using two different nitrification pathways are shown in Figure S7, suggesting that the model is not sensitive to variabilities in reaction stoichiometry. This is due to the inherent electron balance of redox reaction system. Intuitively, while using NO_2^- for denitrification allows the same amount of organic carbon to remove more N (as N_2) compared to using NO_3^- (1C: 4/3N vs 1C:4/5N; Table S1), the production of NO_2^- via nitrification also consumes less oxygen (Table S1). The oxygen saved is then available for aerobic organic matter degradation, reducing the pool of leftover organic matter available for denitrification. This creates a compensatory effect that stabilizes the overall outcome.

SI.5 Data sources from the literature and the treatment of the data

1. Pearl River Estuary region: Sediment oxygen uptake (SOU) were taken from our previous study by Sun et al. (2024 and 2025)[1,10]. SOU is measured under sufficient bottom-water O_2 level, thus it can be used as an approximation for the rates of organic carbon mineralization (see SI.1).
2. Northern Gulf of Mexico: Denitrification rates in Figure 7A (in the main text) are taken from Lehrter et al. (2012)[11], and the fluxes of dissolved inorganic carbon (DIC) across the sediment-water interface was used as a substitute for SOU, because SOU can be limited by low oxygen concentrations in the bottom waters. Bottom-water nitrate concentrations and SOU in Figure 7B are from McCarthy et al. (2013 and 2015)[12,13].
3. Gulf of St Lawrence: data from Thibodeau et al. (2010) and Alkhatib et al. (2012)[14,15].
4. The Changjiang Estuary region: SOU, denitrification, and bottom-water nitrate data are from Song et al. (2021)[16]. The original data include results from sediment incubation measurements under bottom waters with various oxygen levels (oxic, ambient, and hypoxia). To account for the underestimation of sediment organic carbon remineralization rate using SOU under low-oxygen conditions (hypoxic conditions), we correct the SOU values using SOU measured under ambient or oxic conditions (Table S2).
5. The Long Island Sound: data from Mazur et al. (2021)[17].
6. The Chesapeake Bay: data from Boynton et al. (2018, 2019, and 2022)[18–20].

Table S1 Reactions in sediments associated with nitrogen cycling. $(CH_2O)_x(NH_3)_y$ represents the average organic matter in the sediments with a C:N ratio of x:y.

**Process**	**Reaction**
Aerobic respiration	$(CH_2O)_x(NH_3)_y + xO_2 \rightarrow xCO_2 + yNH_3 + xH_2O$
Denitrification (from NO_3^-)	$(CH_2O)_x(NH_3)_y + \frac{4}{5}xNO_3^- \rightarrow xCO_2 + yNH_3 + \frac{2}{5}xN_2 + \frac{3}{5}xH_2O + \frac{4}{5}xOH^-$
Denitrification (from NO_2^-)	$(CH_2O)_x(NH_3)_y + \frac{4}{3}xNO_2^- \rightarrow xCO_2 + yNH_3 + \frac{2}{3}xN_2 + \frac{4}{3}xH_2O + \frac{4}{3}xOH^-$
Nitrification	$NH_4^+ + 2O_2 \rightarrow NO_3^- + H_2O + 2H^+$
Nitrification (incomplete)	$NH_4^+ + \frac{3}{2}O_2 \rightarrow NO_2^- + H_2O + 2H^+$
Anaerobic oxidation of ammonium	$NH_4^+ + NO_2^- \rightarrow N_2 + 2H_2O$
Dissimilatory nitrate reduction to ammonium	$xNO_3^- + 2(CH_2O)_x(NH_3)_y \rightarrow (x+2y)NH_3 + 2xCO_2 + xOH^-$

Table S2 Sampling site, location, total water depth (depth), the ratio of organic carbon and nitrogen remineralization in surface sediments ($r_{C:N}$), sediment oxygen diffusive flux across the sediment-water interface (SWI) ($F_{O_2}^{diff}$), bioirrigation flux of ammonium and nitrate across the SWI ($F_{NH_4^+}^{irr}$ and $F_{NO_3^-}^{irr}$), and total flux of ammonium and nitrate ($F_{NH_4^+}$ and $F_{NO_3^-}$) across the SWI. A negative flux number indicates efflux from sediments into the overlying waters.

Sites	Date (mm/dd/yyyy)	Latitude (°N)	Longitude (°E)	Depth (m)	$r_{C:N}$	$F_{O_2}^{diff}$ (mmol m^{-2} d^{-1})	$F_{NH_4^+}^{irr}$ (mmol m^{-2} d^{-1})	$F_{NO_3^-}^{irr}$(mmol m^{-2} d^{-1})	$F_{NH_4^+}$ (mmol m^{-2} d^{-1})	$F_{NO_3^-}$(mmol m^{-2} d^{-1})
A03	06/03/2021	113.741	22.602	15	10.6	19.5	-1.20±0.01	1.73±0.55	-1.74±0.01	2.51±0.80
A05	06/03/2021	113.765	22.463	14	10.5	13.9	-0.705±0.004	0.757±0.001	-1.02±0.01	1.09±0.01
A08	06/04/2021	113.788	22.266	9	10.8	10.8	-0.786±0.016	0.264±0.123	-1.14±0.02	0.382±0.178
A11	06/04/2021	113.866	22.093	21	8.8	21.9	-3.83±0.72	0.040±0.001	-5.54±1.03	0.058±0.002
F101	06/07/2021	113.128	21.806	13	8.1	12.1	-1.15±0.02	0.152±0.001	-1.66±0.03	0.220±0.001
F103	06/07/2021	113.189	21.694	29	6.9	9.20	-0.596±0.338	-0.011±0.001	-0.862±0.489	-0.015±0.002
F201	06/08/2021	113.389	21.914	12	9.0	15.4	-2.10±0.04	0.182±0.001	-3.04±0.06	0.263±0.001
P204	06/19/2021	113.052	21.584	33	9.3	13.7	-0.755±0.284		-1.09±0.41	
F204	06/20/2021	113.456	21.744	33	6.6	6.33	-0.502±0.130	-0.027±0.001	-0.726±0.188	-0.040±0.001
F301	06/09/2021	113.548	21.990	11	7.3	16.1	-5.40±0.89	0.239±0.001	-7.81±1.29	0.346±0.001
F603	06/22/2021	114.085	22.037	31	9.3	11.1	-1.09±0.47	-0.002±0.001	-1.57±0.68	-0.002±0.001
J103	06/18/2021	112.738	21.431	36	6.7	9.80	-1.07±0.18	-0.083±0.001	-1.54±0.25	-0.120±0.001
P101	06/05/2021	112.488	21.581	16	9.4	7.72	-1.30±0.25	0.004±0.001	-1.88±0.36	0.006±0.001
2A01b	07/08/2021	114.123	21.751	41	8.6	6.41	-0.180±0.027	-0.084±0.042	-0.260±0.039	-0.122±0.061
2A02	07/04/2021	114.252	21.501	63	6.4	5.32	-0.470±0.040	-0.120±0.001	-0.680±0.058	-0.173±0.001
2A01	07/01/2021	113.998	22.001	33	7.5	13.4	-1.25±0.52	-0.211±0.001	-1.80±0.75	-0.305±0.001
2P03	07/06/2021	113.851	21.997	24	10.2	3.52	-0.907±0.018	-0.042±0.020	-1.31±0.03	-0.061±0.029
2P02	07/06/2021	113.698	21.994	16	10.1	3.96	-1.50±0.11	-0.013±0.004	-2.15±0.02	-0.019±0.006
F702	06/22/2021	114.212	22.070	31	6.7		-1.62±0.38	0.007±0.001	-2.33±0.55	0.010±0.001
**Average**					**8.5±1.5**	**14.5±6.9**	**-1.38±1.28**	**0.164±0.460**	**-1.99±1.86**	**0.237±0.665**

Table S3 Data for other coastal oceans under seasonal hypoxia.

Region	Station	Bottom O_2 (μmol L^{-1})	Bottom NO_3^- (μmol L^{-1})	SOU (mmol m^{-2} d^{-1})	Corrected SOU (mmol m^{-2} d^{-1})	F_{N2} (mmol m^{-2} d^{-1})	Other information	Reference
Changjiang Estuary	C1	16	18.6	0	19	0.73	Aug_hyp	[16]
Changjiang Estuary	F2	16	15.6	2	13.25	0.48	Aug_hyp	[16]
Changjiang Estuary	F1	84.8	8.6	6	14	0.81	Aug_ambient	[16]
Changjiang Estuary	F1	16	8.6	0	14	0.54	Aug_hyp	[16]
Changjiang Estuary	O11	87.1	15.6	5	9.5	0.72	Aug_ambient	[16]
Changjiang Estuary	O11	16	15.6	2.5	9.5	0.515	Aug_hyp	[16]
Northern Gulf of Mexico	Z01b	51.9	13.7	6.91	21.5	0.00	Apr	[11]
Northern Gulf of Mexico	Z02b	60.2	7.2	7.04	15	0.18	Apr	[11]
Northern Gulf of Mexico	Z03b	67.9	1.5	2.15	7	0.17	Apr	[11]
Northern Gulf of Mexico	Z01b	135.4	1.7	6.91	20	1.34	Jun	[11]
Northern Gulf of Mexico	Z02b	11.5	8.6	7.04	19	0.20	Jun	[11]
Northern Gulf of Mexico	Z03b	137.9	1.9	2.15	18	0.88	Jun	[11]
Northern Gulf of Mexico	Z01b	142.5	4.7	6.91	15	0.27	Sep	[11]
Northern Gulf of Mexico	Z02b	16.0	8.4	7.04	14	0.40	Sep	[11]
Northern Gulf of Mexico	Z03b	118.4	5.6	2.15	18	0.25	Sep	[11]
Northern Gulf of Mexico	Z02b	131.8	1.8	7.04	10	0.94	Apr	[11]
Northern Gulf of Mexico	Z03b	140.2	1.1	2.15	15	0.19	Apr	[11]
Northern Gulf of Mexico	Z04b	129.0	1.0	5.47	18		Apr	[11]
Northern Gulf of Mexico	Z02b	26.4	6.9	7.04	12	0.49	Aug	[11]
Northern Gulf of Mexico	Z03b	15.8	2.5	2.15	18	1.80	Aug	[11]
Northern Gulf of Mexico	Z04b	106.3	0.9	5.47	20	1.23	Aug	[11]
Northern Gulf of Mexico	B7	3.4375	7.81	26.1			Aug_2009	[12,13]
Northern Gulf of Mexico	B7	50.625	1.87	43.2			May_2010	[12,13]
Northern Gulf of Mexico	B7	4.6875	9.56	15.0			May_2011	[12,13]
Northern Gulf of Mexico	CT2	27.1875	10.5	20.4			May_2010	[12,13]
Northern Gulf of Mexico	F5	59.0625	7.89	16.4			May_2010	[12,13]
Northern Gulf of Mexico	MRM	9.0625	7.96	21.3			Aug_2009	[12,13]
Northern Gulf of Mexico	MRM	28.4375	8.65	34.3			May_2010	[12,13]
Gulf of St Lawrence	E5	65	25	6.41				[14,15]
Gulf of St Lawrence	24	65	25	6.49				[14,15]
Gulf of St Lawrence	23	63	20	4.3				[14,15]
Gulf of St Lawrence	21	75	22	3.22				[14,15]
Gulf of St Lawrence	19	108	22	3.75				[14,15]
Gulf of St Lawrence	18	123	22	4.95				[14,15]
Gulf of St Lawrence	TCA	106	25	4.36				[14,15]
Gulf of St Lawrence	TCE	94	25	2.31				[14,15]
Gulf of St Lawrence	EXR	138	2.5	18.48			Summer	[17]
Gulf of St Lawrence	WLIS	172	3.09	13.2			Summer	[17]
Gulf of St Lawrence	ARTG	112	2.64	9.24			Summer	[17]
Gulf of St Lawrence	ELIS	222	1.32	9.12			Summer	[17]

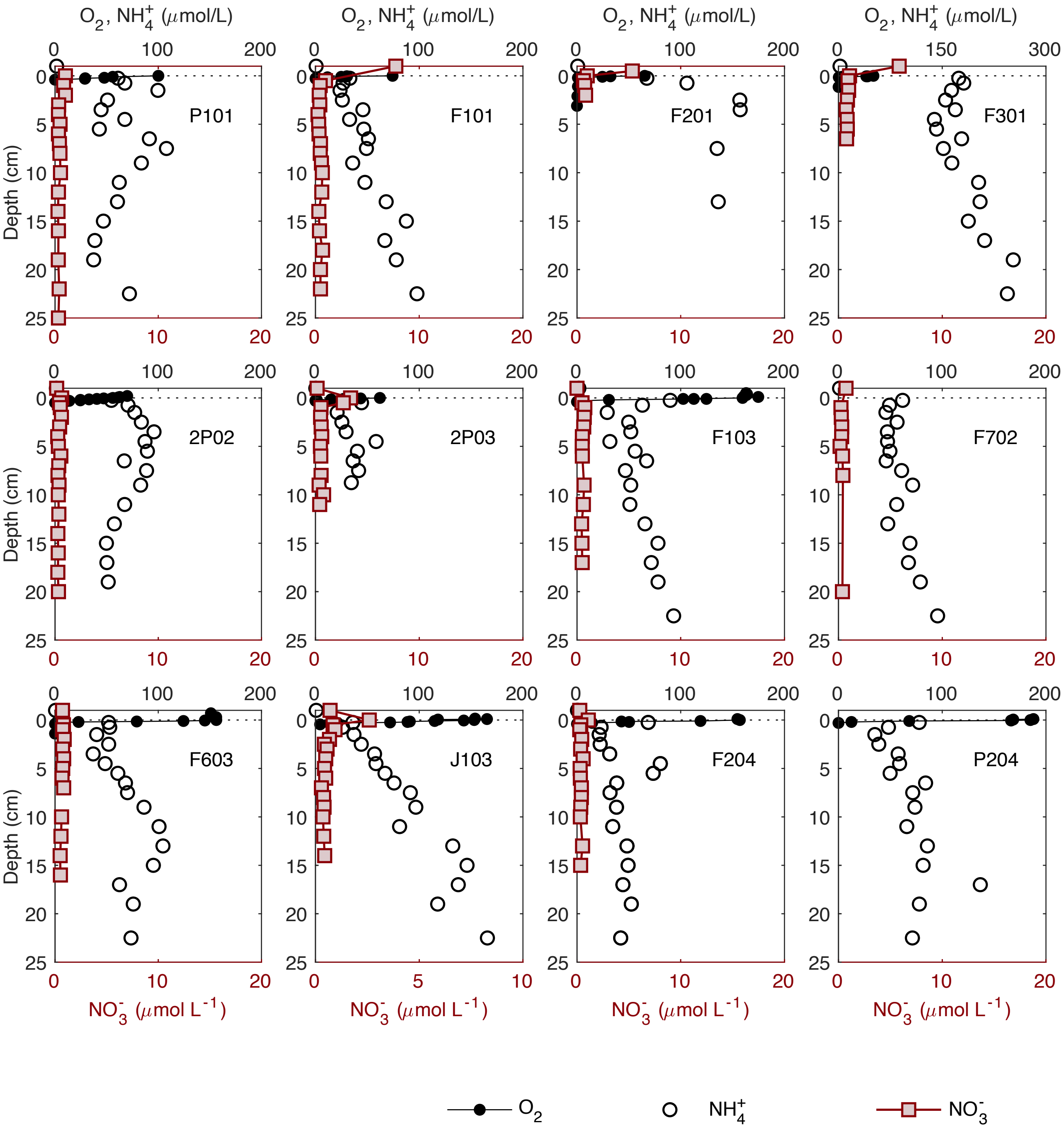

Figure S1 Vertical distributions of oxygen (O_2), nitrate (NO_3^-), and ammonium (NH_4^+) in the sediments (see Fig. 2 in the main text for profiles from sampling sites across the estuarine salinity gradient).

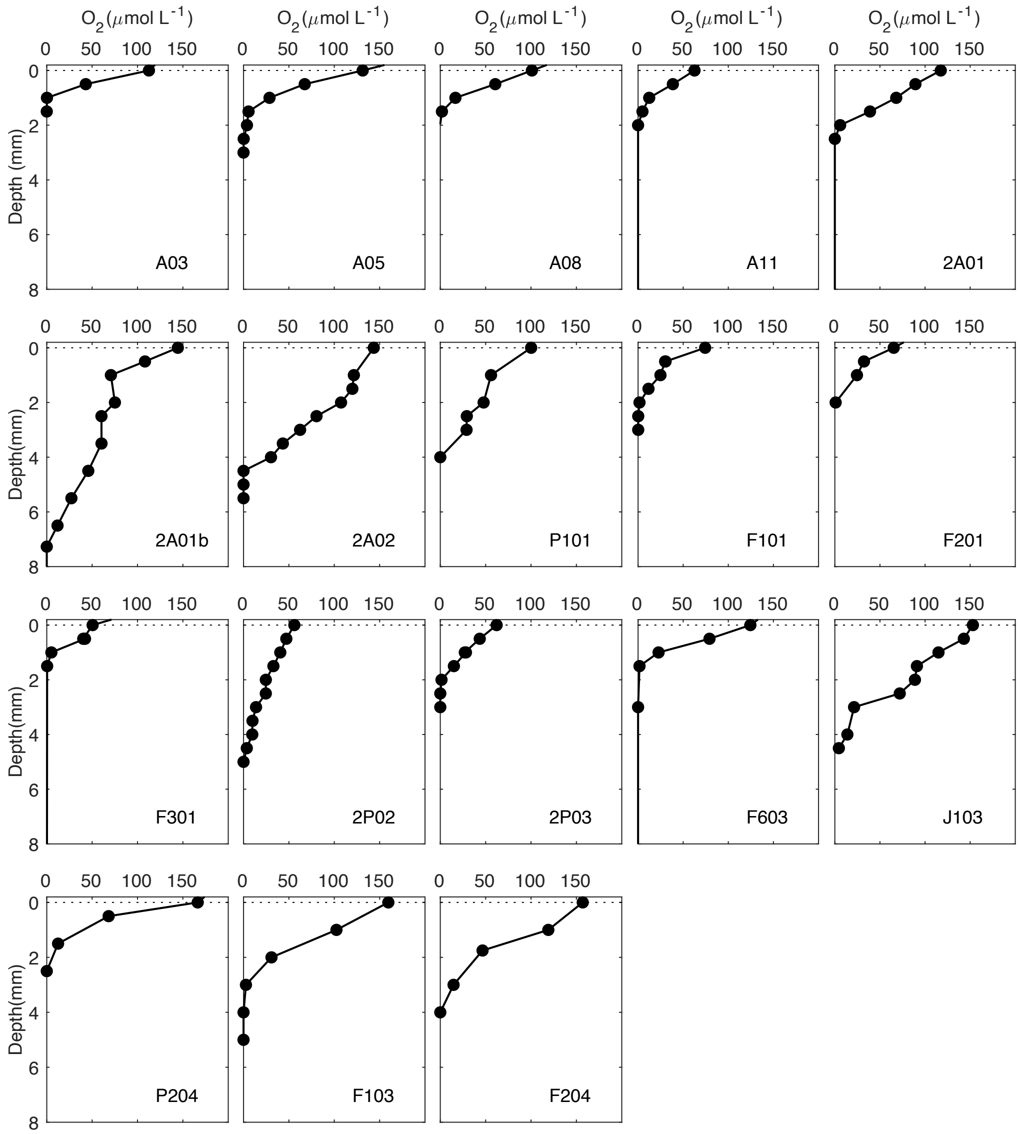

Figure S2 Vertical distributions of oxygen in the sediments.

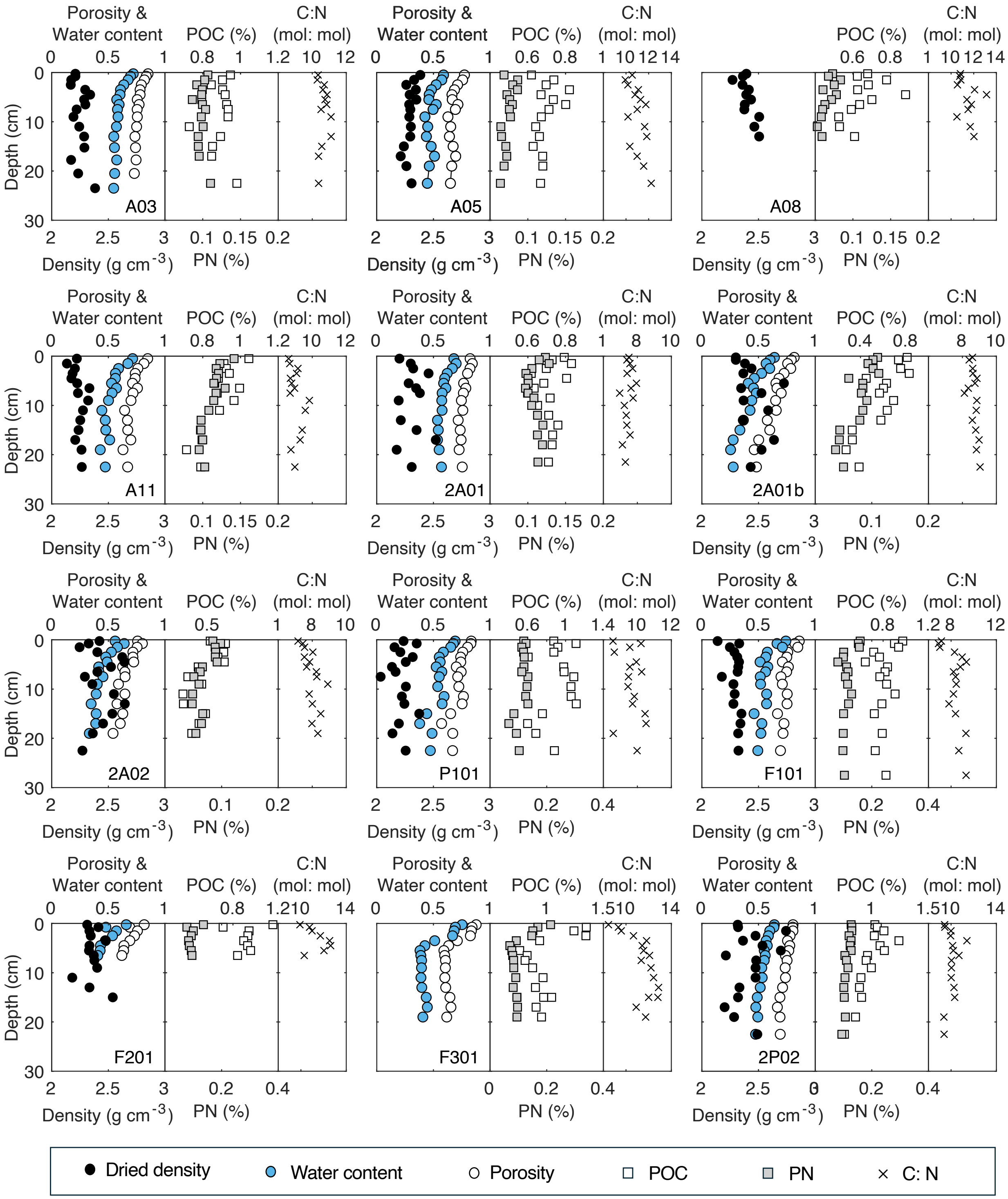

Figure S3 Vertical profiles of sediment dried density, porosity, particulate organic carbon (POC), particulate nitrogen (PN), and the ratios of POC and PN (C:N).

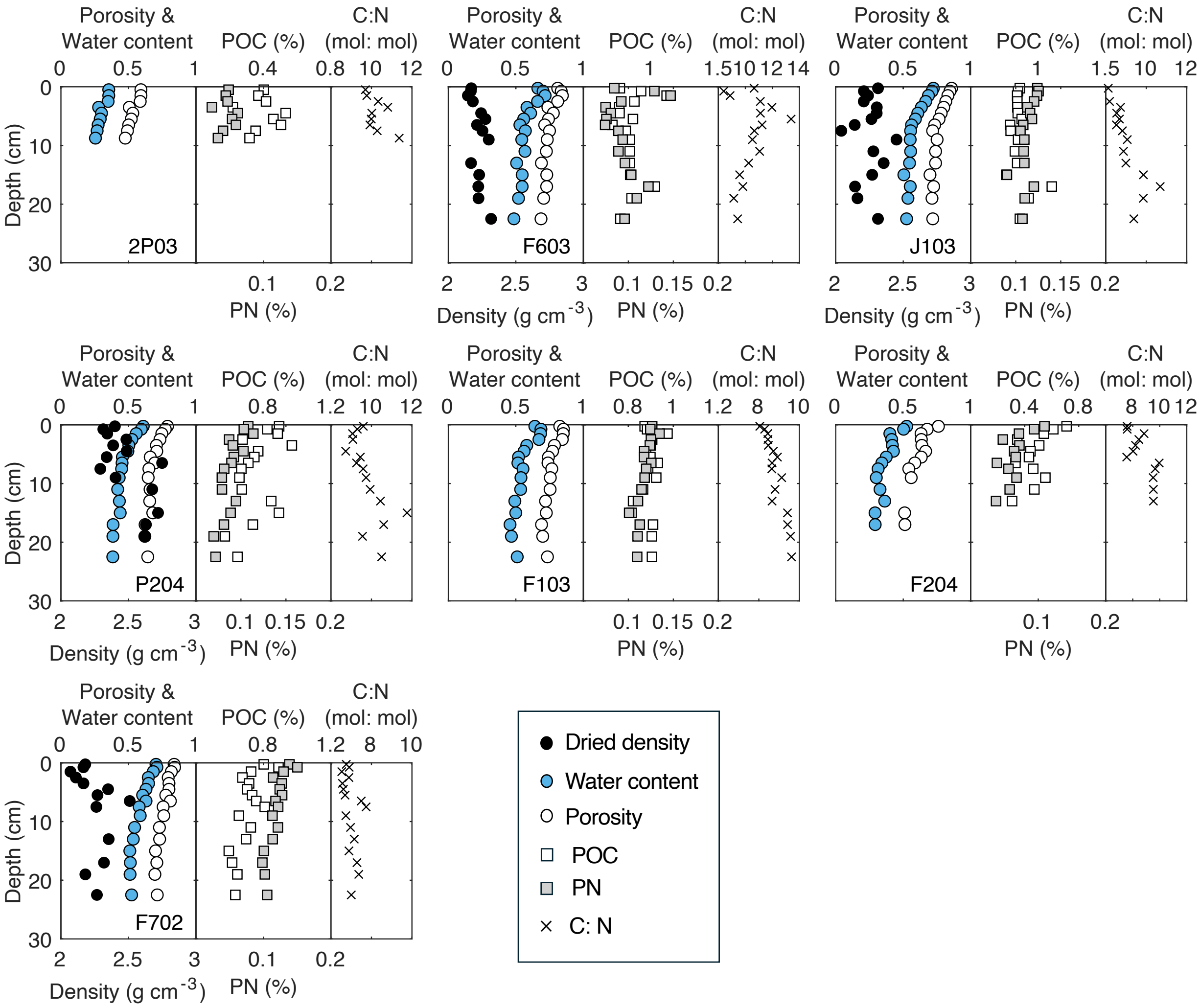

Figure S3 (continued) Vertical profiles of sediment dried density, porosity, particulate organic carbon (POC), particulate nitrogen (PN), and the ratio of POC and PN (C:N).

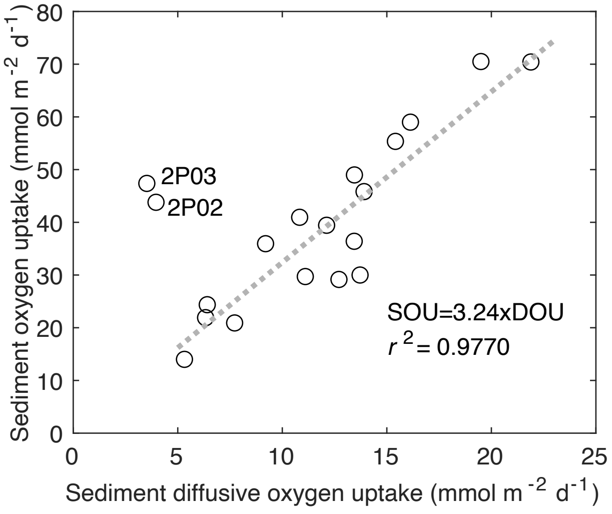

Figure S4 Sediment oxygen uptake (SOU, measured by incubation) vs sediment diffusive oxygen uptake (DOU, calculated from oxygen profiles using Fick's Law). The sediment oxygen profiles at sites 2P02 and 2P03 were conducted during bad weather (rough sea conditions), which caused the sensor signals to take longer to stabilize. We suspect that the oxygen profiles were smoothed due to disturbances in the sediments by the sensor, leading to measured concentration gradients that might be substantially lower than the in-situ values. Consequently, the DOU may have been underestimated. We exclude these two sites from the linear fit (dotted line).

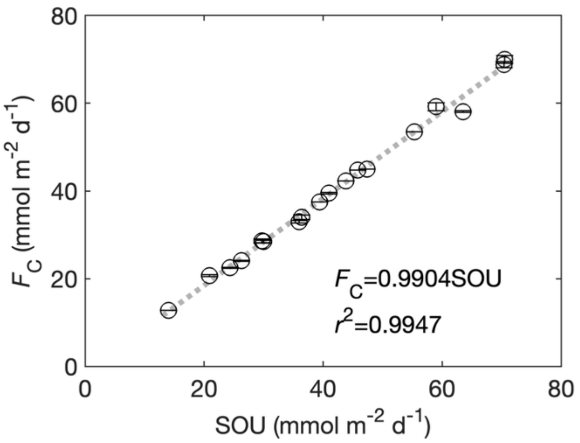

Figure S5 The rates of organic carbon remineralization in sediments (F_C) versus sediment oxygen uptake (SOU)

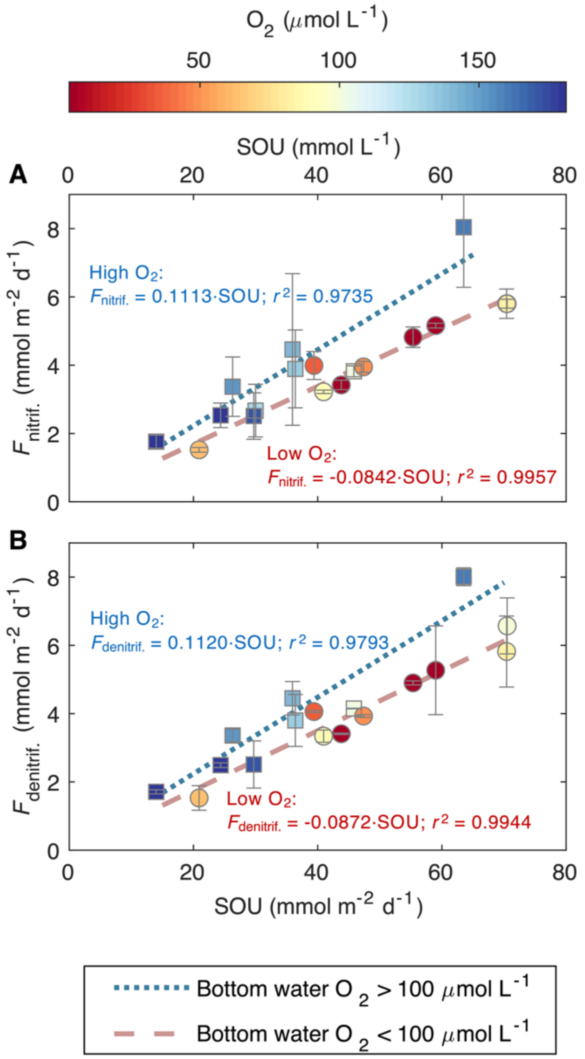

Figure S6 Sediment nitrification and denitrification rates versus sediment oxygen uptake in the Pearl River Estuary region. The rates were estimated using molecular diffusive fluxes of nitrate and ammonium.

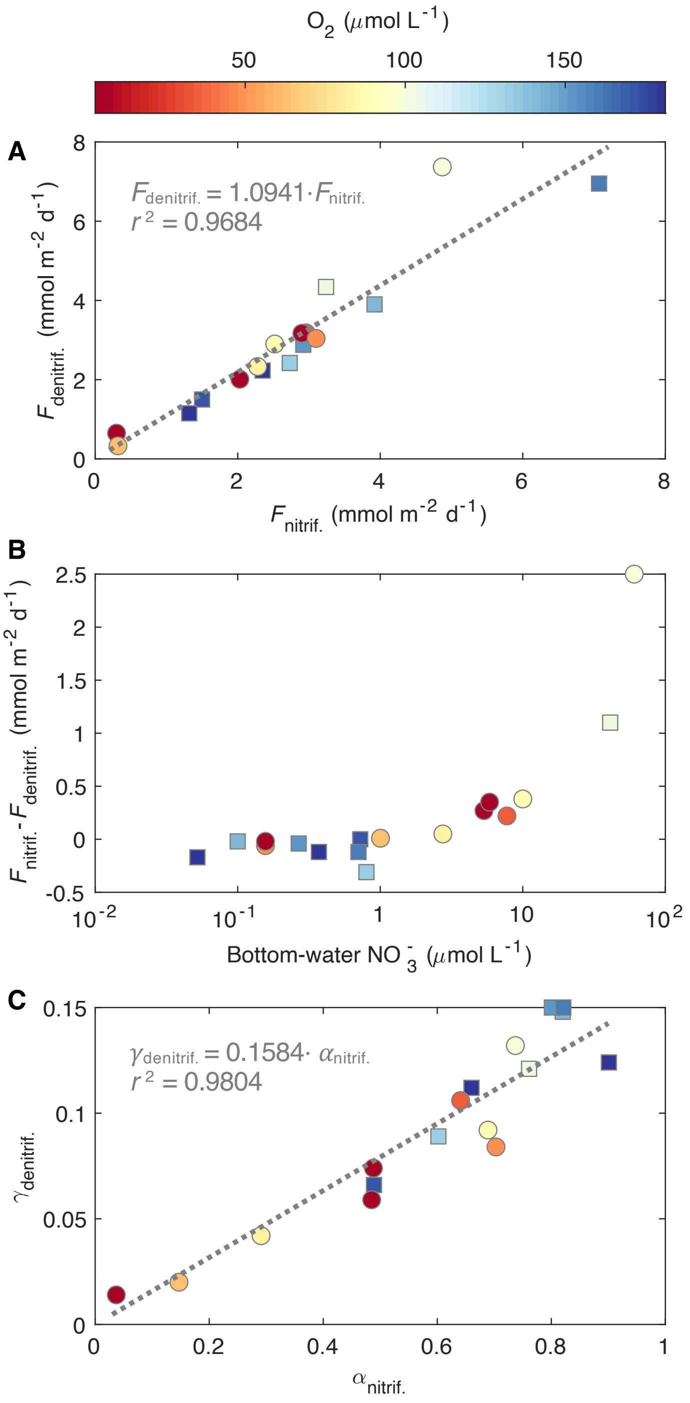

Figure S7 (**A**) Sediment denitrification rate vs nitrification rate, (**B**) the difference between nitrification and denitrification rates vs bottom water nitrate concentration, and (**C**) the proportion of organic matter remineralization contributed by denitrification ($\gamma_{denitrif.}$) vs the proportion of remineralized ammonium that is oxidized via nitrification in the sediments ($\alpha_{nitrif.}$)

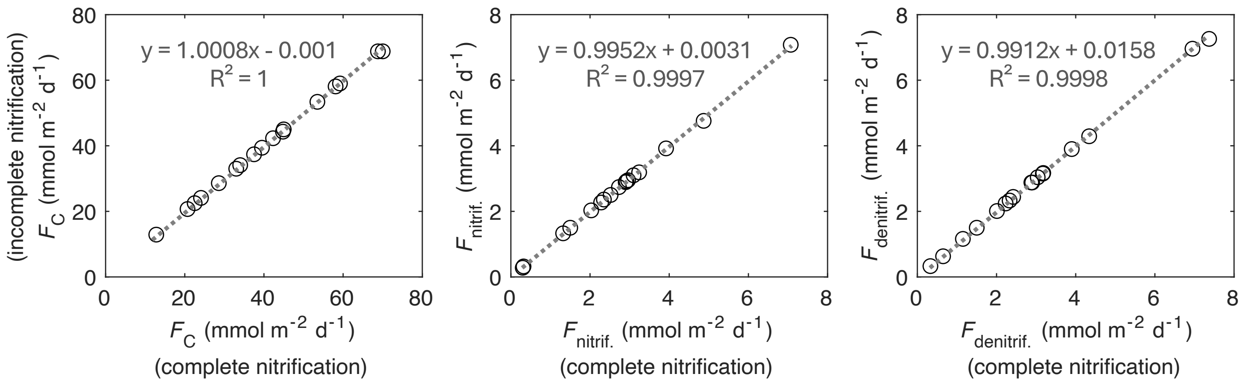

Figure S8 Rates organic carbon remineralization, nitrification, and denitrification estimated using models with incomplete nitrification vs complete nitrification.

Reference

(1) Sun, J.; Yu, L.; Yang, X.; Gan, J.; Yin, H.; Li, J. Sediment Oxygen Uptake and Hypoxia in Coastal Oceans, the Pearl River Estuary Region. *Water Res.* **2024**, *267*, 122499. https://doi.org/10.1016/j.watres.2024.122499.

(2) Murrell, M. C.; Lehrter, J. C. Sediment and Lower Water Column Oxygen Consumption in the Seasonally Hypoxic Region of the Louisiana Continental Shelf. *Estuaries Coasts* **2011**, *34* (5), 912–924. https://doi.org/10.1007/s12237-010-9351-9.

(3) Christensen, J. P.; Devol, A. H.; Smethie, W. M. Biological Enhancement of Solute Exchange between Sediments and Bottom Water on the Washington Continental Shelf. *Cont. Shelf Res.* **1984**, *3* (1), 9–23. https://doi.org/10.1016/0278-4343(84)90040-2.

(4) Katsev, S.; Chaillou, G.; Sundby, B.; Mucci, A. Effects of Progressive Oxygen Depletion on Sediment Diagenesis and Fluxes: A Model for the Lower St. Lawrence River Estuary. *Limnol Oceanogr* **2007**, *52* (6), 2555–2568. https://doi.org/10.4319/lo.2007.52.6.2555.

(5) Meile, C.; Berg, P.; Cappellen, P. V.; Tuncay, K. Solute-Specific Pore Water Irrigation: Implications for Chemical Cycling in Early Diagenesis. *J. Mar. Res.* **2005**, *63* (3), 601–621. https://doi.org/10.1357/0022240054307885.

(6) Mackin, J. E.; Aller, R. C. Ammonium Adsorption in Marine Sediments1. *Limnol. Oceanogr.* **1984**, *29* (2), 250–257. https://doi.org/10.4319/lo.1984.29.2.0250.

(7) Ip, C. C. M.; Li, X. D.; Zhang, G.; Farmer, J. G.; Wai, O. W. H.; Li, Y. S. Over One Hundred Years of Trace Metal Fluxes in the Sediments of the Pearl River Estuary, South China. *Environ Pollut* **2004**, *132* (1), 157–172. https://doi.org/10.1016/j.envpol.2004.03.028.

(8) Jia, G.; Xu, S.; Chen, W.; Lei, F.; Bai, Y.; Huh, C. A. 100-Year Ecosystem History Elucidated from Inner Shelf Sediments off the Pearl River Estuary, China. *Mar Chem* **2013**, *151*, 47–55. https://doi.org/10.1016/j.marchem.2013.02.005.

(9) Zhou, L. Sediment Oxygen Uptake and Carbon Mineralization in the Pearl River Estuary and Adjacent Coastal Waters. **2022**. https://doi.org/10.14711/thesis-991013088359403412.

(10) Sun, J. Sediment Biogeochemical Cycles and Exchanges with the Water Column in the Coastal Ocean around the Pearl River Estuary, PhD Thesis, The Hong Kong University of Science and Technology. doi: 10.14711/thesis-991013384163903412, 2024.

(11) Lehrter, J. C.; Beddick, D. L.; Devereux, R.; Yates, D. F.; Murrell, M. C. Sediment-Water Fluxes of Dissolved Inorganic Carbon, O2, Nutrients, and N2 from the Hypoxic Region of the Louisiana Continental Shelf. *Biogeochemistry* **2012**, *109* (1–3), 233–252. https://doi.org/10.1007/s10533-011-9623-x.

(12) McCarthy, M. J.; Newell, S. E.; Carini, S. A.; Gardner, W. S. Denitrification Dominates Sediment Nitrogen Removal and Is Enhanced by Bottom-Water Hypoxia in the Northern Gulf of Mexico. *Estuaries Coasts* **2015**, *38* (6), 2279–2294. https://doi.org/10.1007/s12237-015-9964-0.

(13) McCarthy, M. J.; Carini, S. A.; Liu, Z.; Ostrom, N. E.; Gardner, W. S. Oxygen Consumption in the Water Column and Sediments of the Northern Gulf of Mexico Hypoxic Zone. *Estuar Coast Shelf Sci* **2013**, *123*, 46–53. https://doi.org/10.1016/j.ecss.2013.02.019.

(14) Alkhatib, M.; Lehmann, M. F.; Giorgio, P. A. del. The Nitrogen Isotope Effect of Benthic Remineralization-Nitrification-Denitrification Coupling in an Estuarine Environment. *Biogeosciences* **2012**, *9* (5), 1633–1646. https://doi.org/10.5194/bg-9-1633-2012.

(15) Thibodeau, B.; Lehmann, M. F.; Kowarzyk, J.; Mucci, A.; Gélinas, Y.; Gilbert, D.; Maranger, R.; Alkhatib, M. Benthic Nutrient Fluxes along the Laurentian Channel: Impacts on the N Budget of the St. Lawrence Marine System. *Estuar., Coast. Shelf Sci.* **2010**, *90* (4), 195–205. https://doi.org/10.1016/j.ecss.2010.08.015.

(16) Song, G.; Liu, S.; Zhang, J.; Zhu, Z.; Zhang, G.; Marchant, H. K.; Kuypers, M. M. M.; Lavik, G. Response of Benthic Nitrogen Cycling to Estuarine Hypoxia. *Limnol Oceanogr* **2021**, *66* (3), 652–666. https://doi.org/10.1002/lno.11630.

(17) Mazur, C. I.; Al-Haj, A. N.; Ray, N. E.; Sanchez-Viruet, I.; Fulweiler, R. W. Low Denitrification Rates and Variable Benthic Nutrient Fluxes Characterize Long Island Sound Sediments. *Biogeochemistry* **2021**, *154* (1), 37–62. https://doi.org/10.1007/s10533-021-00795-7.

(18) Boynton, W. R.; Ceballos, M. A. C.; Hodgkins, C. L. S.; Liang, D.; Testa, J. M. Large-Scale Spatial and Temporal Patterns and Importance of Sediment–Water Oxygen and Nutrient Fluxes in the Chesapeake Bay Region. *Estuaries Coasts* **2022**, 1–20. https://doi.org/10.1007/s12237-022-01127-0.

(19) Boynton, W. R.; Ceballos, M. A. C.; Bailey, E. M.; Hodgkins, C. L. S.; Humphrey, J. L.; Testa, J. M. Oxygen and Nutrient Exchanges at the Sediment-Water Interface: A Global Synthesis and Critique of Estuarine and Coastal Data. *Estuaries Coasts* **2018**, *41* (2), 301–333. https://doi.org/10.1007/s12237-017-0275-5.

(20) Boynton, W.; Ceballos, M. A. C. Chesapeake Bay and Maryland Coastal Bays Sediment-Water Oxygen and Nutrient Flux Data Set. *Mendeley Data* **2019**, No. V1. https://doi.org/10.17632/jpwvc5jytk.1.

(21) Laursen, A. E.; Seitzinger, S. P. The Role of Denitrification in Nitrogen Removal and Carbon Mineralization in Mid-Atlantic Bight Sediments. *Cont. Shelf Res.* **2002**, *22* (9), 1397–1416. https://doi.org/10.1016/s0278-4343(02)00008-0.

(22) Seitzinger, S. P.; Giblin, A. E. Estimating Denitrification in North Atlantic Continental Shelf Sediments. *Biogeochemistry* **1996**, *35* (1), 235–260. https://doi.org/10.1007/bf02179829.
